# Revision of the pseudo-orbweavers of the genus *Fecenia* Simon, 1887 (Araneae, Psechridae), with emphasis on their pre-epigyne

**DOI:** 10.3897/zookeys.153.2110

**Published:** 2011-12-09

**Authors:** Steffen Bayer

**Affiliations:** 1 Senckenberg Research Institute, Senckenberganlage 25, 60325 Frankfurt am Main, Germany

**Keywords:** Taxonomy, copulatory organs, spination, distribution, South-East Asia, pseudo-orbweb, enrolled leaf, predatory behaviour, mating behaviour, moult, identification key

## Abstract

The present paper provides a taxonomic revision of the genus *Fecenia* with emphasis on the characteristics of the pre-epigynes which are integrated for the first time into an identification key. As a result, one species is revalidated, *Fecenia protensa* Thorell, 1891, **stat. n.**, and two new junior synonyms for *Fecenia protensa* are recognised: *Fecenia sumatrana* Kulczyński, 1908, **syn. n.** and *Fecenia nicobarensis* (Tikader, 1977), **syn. n**. New records are reported: *Fecenia ochracea* (Doleschall, 1859)from Malaysian Borneo, *Fecenia macilenta* (Simon, 1885) from Sumatra, Indonesia, *Fecenia protensa* from Thailand and Malaysia, *Fecenia travancoria* Pocock, 1899 from Sri Lanka and Thailand, and *Fecenia cylindrata* Thorell, 1895 from Thailand and Laos. Additional information on the biology of *Fecenia* is provided and the validity of characters for identifying *Fecenia* species is discussed.

## Introduction

Representatives of the spider genus *Fecenia* are distributed from southern India to the Solomon Islands. They are not known beyond the latitudes of 25°N and 15°S. To date (Platnick 2011) this genus comprises five valid species. *Fecenia* species possess relatively long and prograde legs. The first two pairs are directed anteriorly whereas the third and fourth leg pairs are directed posteriorly. *Fecenia* species have a flat carapace and a slender body shape (Thorell 1881). Their chelicerae are short and strong and bear a distinct condyle latero-proximally (Levi 1982). Adults build a vertical web, which is similar to the typical orbwebs of (most) Araneidae and related families like Tetragnathidae, respectively. Thus representatives of *Fecenia* are here called ‘pseudo-orbweavers'. Simon (1892) described their web as more irregular than the webs of Araneidae. Furthermore he stated that it contains an enrolled leaf as a retreat in the centre. Despite this somewhat similar web style, *Fecenia* is not closely related to the Araneidae and does not belong to the Orbiculariae either (Coddington 1990). Together with *Psechrus* Thorell, 1878, this genus belongs to the Psechridae Simon, 1890 (Simon 1892; Lehtinen 1967; Levi 1982; Griswold 1993; Griswold et al. 2005; Platnick 2011). Previously the pseudo-orbweavers were revised twice. Levi (1982) provided a worldwide revision and Wang and Yin (2001) covered Chinese representatives. In the study of Lehtinen (1967) several *Fecenia* species were synonymised. Levi (1982) matched a female of a different species with the male of *Fecenia macilenta* (Simon, 1885). Murphy (1986) recognised this mistake and described the female of *Fecenia macilenta* for the first time. At present, further taxonomic ambiguities still persist. Some of these were caused by descriptions of new species using subadult females (which only possess pre-epigynes) as type specimens.

Pre-epigynes do not occur in all entelegyne spiders, but seem to be common within the families supposed to be related to Psechridae (Griswold 1993; Griswold et al. 1999, 2005), e.g. Pisauridae, Lycosidae, Stiphidiidae, Zoropsidae and Ctenidae. Up to now pre-epigynes were mostly disregarded in arachnological studies. There are some first descriptions where pre-epigynes had been erroneously regarded as epigynes (e.g. *Psechrus mimus* Chamberlin, 1924, *Heteropoda shillongensis* Sethi and Tikader, 1988, *Psechrus ghecuanus* Thorell, 1897). A study on *Agelena labyrinthica* (Clerck, 1757) noted the presence of a primordial copulatory organ in females (Strand 1906). Jäger and Ono (2000), Jäger (2008) as well as Jäger and Bayer (2009) illustrated pre-epigynes of a few particular species of *Olios* Walckenaer, 1837 and *Heteropoda* Latreille, 1804 respectively. Several differently developed primordial copulatory organs in different stages of immature females of *Cupiennius salei* (Keyserling, 1877) were documented in Lachmuth et al. (1985). In Psechridae pre-epigynes were illustrated for the first time by Levi (1982). However, he studied only a few species in this regard. Moreover, in the case of *Psechrus himalayanus* Simon, 1906, he regarded a pre-epigyne as an adult epigyne. This led to misunderstandings in species determination and characterisation. As an ongoing revision shows (Bayer unpubl. data), Griswold (1993) examined a subadult female of *Psechrus marsyandi* Levi, 1982, identified as *Psechrus himalayanus*, as the female representative of the genus *Psechrus* in his study on the phylogeny of Lycosoidea. Wang and Yin (2001) showed the pre-epigyne of one species, *Psechrus rani* Wang and Yin, 2001, and compared it with features of the conspecific adult female. A fairly complete investigation on the pisaurid genus *Thalassius* Simon, 1885 was carried out by Sierwald (1987) where most species concerned were characterised by their pre-epigyne II (penultimate instars) and some even by their pre-epigyne I (antepenultimate instars). An even more detailed study on American Pisauridae described changes in the development of pre-epigynes of different stages via very detailed illustrations (Sierwald 1989). Nevertheless, no study to date has examined variation within pre-epigynes of penultimate instar females within a species, nor has there been any attempt to integrate the pre-epigyne and pre-vulva- features into an identification key. In this context, the intention of this paper is to provide a thorough taxonomic revision of *Fecenia* including some remarks on their biology and above all the character states of pre-epigynes.

## Material and methods

Part of the spider material was collected by hand during an expedition in Thailand and Laos from October-December 2009. Further material was obtained from colleagues, who collected specimens in different regions of SE Asia. Most of the material examined in the present study was borrowed from several natural history museums, which are listed below. Examinations and illustrations were made using a Leica MZ 165 C stereomicroscope with a drawing mirror. Photos of living spiders were taken with a Canon EOS 500D (equipped with a Sigma 105 macro lens and a Canon ringlite). Photos of preserved spiders and copulatory organs were taken with a Sony DSC W70 compact camera via the ocular of the stereomicroscope. The material was preserved in 70% denatured ethanol. Female copulatory organs were cleared from surrounding hairs and dissected. The opaque tissue surrounding the vulva was removed. Vulvae were cleared in 96% DL-lactic acid (C_3_H_6_O_3_). As the cuticle surrounding the epigyne may curl and structures may get shifted in the course of applying lactic acid, this method could not be applied to every specimen. In males, hairs along the margin of the cymbium were removed to give a clear view of the bulb structures.

All measurements are in millimetres (mm). Leg formula (from longest to shortest leg) and leg spination pattern follow those in Bayer and Jäger (2010). In leg/palp spination the femur, patella, tibia and metatarsus (tarsus in palp) are listed in exactly this sequence. First, all spines on the prolateral surface of the respective limb article are counted and listed, then the ones on the dorsal, then retrolateral and finally the ventral surface. Thus the resulting number is generally one of 4-digits. Some limb articles, e.g. the femur and patella, always lack ventral spines, so here the number is of 3-digits. If a spination pattern from a certain limb article differs between the left and right sides, the pattern for the right article is listed in parenthesis behind, without a blank. Palp and leg lengths are listed as: total (femur, patella, tibia, metatarsus, tarsus).

Abbreviations used in the text: ALE – Anterior lateral eye. AME – Anterior median eye. AML – Anterior margin of lateral lobe. AS – Anterior part of median septum. bEA – Basal embolus apophysis. BL – Borderline between SSI and TSI. C – Conductor. CA – Cymbium alveolus. CO – Copulatory opening. DRTA – Dorso–retrolateral tibial apophysis. E – Embolus. EF – Epigynal field. EM – Epigynal muscle sigilla. FD – Fertilisation duct. juv. – Juvenile (convention in the present work: juveniles are immature specimens of instars where no sex determination is possible, otherwise called juvenile male or juvenile female). LL – Lateral lobe. MA – Median apophysis. MP – Membranous process of tegulum. PLE – Posterior lateral eye. PME – Posterior median eye. PS – Posterior part of median septum. p.s.a. – Pre-subadult. RPA – Retrolateral patellar apophysis. RTA – Retrolateral tibial apophysis. s.a. – Subadult. SB – Serial individual numbers of Psechridae examined by the author. SH – Spermathecal head. SO – Slit sense organ. SSI – Strongly sclerotised section of internal duct system. T – Tegulum. TR – Transverse edge/ridge of median septum. TSI – Transparent section of internal duct system. VPA – Ventral patellar apophysis.

Terminology of structures belonging to the copulatory organs is given as follows:

The female epigyne consists of two slits, which separate the lateral lobes (LL) from the median septum. The latter is folded transversely, resulting in a transverse edge or ridge (TR) (Fig. 79). Consequently, an anterior part of the septum (AS) and a posterior part (PS) can be distinguished (Fig. 79). Anteriorly, each of the LL exhibits a more or less sclerotised margin (anterior margin of lateral lobe, AML). The entire epigyne is surrounded by an epigynal field (EF), which is a sclerotised area. It is not as intensively sclerotised as the median septum or the LL and is distinguished from the adjacent areas of the ventral opisthosoma by a darker colour. The following structures certainly do not belong to the epigyne, but they may be of additional taxonomic information, so they are illustrated and described here, too. Namely the two muscle sigilla (epigynal muscle sigilla, EM) in front of EF (sometimes they are integrated into the epigynal field) and the slit sense organs (SO) near the epigyne (Fig. 79). The vulva consists of an internal duct system (more precisely a folded slit system, cf. Sierwald 1987). It is divided into an initial, rather transparent section (TSI), a strongly sclerotised section (SSI) and the fertilisation duct (FD) (Fig. 83). The border line (BL) between TSI and SSI is clearly visible (Fig. 83). The initial section of SSI features a wide area with pores leading to associated glands. As this area is presumably homologous to the spermathecal head in *Psechrus* (for location of the spermathecal head see Wang and Yin 2001 or Bayer and Jäger 2010) the term spermathecal head (SH) is used here for *Fecenia* too, despite its different shape (Fig. 83). Griswold (1993: p. 21) even denominated the entire SSI as “head of spermatheca”, which is not followed here. In *Fecenia* it is very difficult to locate the receptaculum. It is not clear where the functional copulatory duct actually ends. Moreover, nobody has ever observed how far a *Fecenia* embolus penetrates within the internal duct system or where the sperm are finally stored.

Apart from structures of a male palp that are well known in arachnology, e.g. conductor, sperm duct or RTA, the *Fecenia* palp shows a retrolateral patellar apophysis (RPA), a ventral patellar apophysis (VPA) and a membranous process (MP) close to the embolus base (Fig. 8). In one species, *Fecenia macilenta*, an additional large apophysis arises dorso-retrolaterally from the tibia (dorso-retrolateral tibial apophysis, DRTA, Fig. 53). Presently it cannot be clarified whether this apophysis is just the dorsal branch of an extended RTA or an additional apophysis. In either case, the DRTA can be regarded as an autapomorphy of this species.

Symbols/styles used in the illustrations: Regular solid lines indicate edges/margins/rims of structures as recognised in the respective view; Weak solid lines indicate edges of fine structures, e.g. membranous structures, or wrinkles in the area of the epigyne; Dashed lines indicate inner walls of ducts and/or slits; Dotted lines (wide) indicate structures shining through the cuticule (e.g. parts of vulva shining through epigynal cuticula). Dotted lines (fine) indicate clear colour differences (e.g. border of epigynal field). In schematic illustrations showing the course of the internal duct system the spermathecal head area is marked with several “T” marks, the copulatory opening with a circle and the end of the fertilisation duct in the direction of the uterus externus with an arrow (see e.g. Fig. 3). When a copulatory opening comprises an elongated slit/area, the circle is put at the central position of that slit/area. Arising points and/or directions of tegular appendages in males are described as clock-positions of the left palp in ventral view. This refers also to directions of some structures of the female vulva. As a convention in this latter case: In every species only the right vulva half is considered.

Museum collections (with curators): AMS – Australian Museum, Sydney (G. Milledge). CAS – California Academy of Sciences, San Francisco (C. E. Griswold, A. Carmichael). HBI – Hunan Biological Research Institute, Hunan Normal University, Changsha (X. J. Peng, L. Ping). IRSN – Institut Royal des Sciences Naturelles de Belgique, Brussels (L. Baert, B. Goddeeris). MCSN – Museo Civico di Storia Naturale, Genoa (M. Tavano). MCZ – Harvard University, Museum of Comparative Zoology, Cambridge, Massachusetts (G. Giribet, L. Leibensperger). MHNG – Muséum d'histoire naturelle, Geneva, Switzerland (P. Schwendinger). MIZ – Museum and Institute of Zoology, Warszawa (D. Mierzwa). MNHN – Muséum National de Histoire Naturelle, Paris (C. Rollard, E. Leguin). NHM – Natural History Museum, London (J. Beccaloni). NHMW – Naturhistorisches Museum Wien, Vienna (J. Gruber, C. Hörweg). NRS – Naturhistoriska Riksmuseet, Stockholm (G. Lindberg). NZSI – Zoological Survey of India, National Zoological Collection, Calcutta. RMNH – Nationaal Natuurhistorisch Museum Naturalis, Leiden, Netherlands (J. Miller, I. J. Smit). SJPC – Sunil Jose Private Collection, Kottayam, India (S. Jose). SMF – Senckenberg Museum, Frankfurt am Main, Germany (P. Jäger, J. Altmann). USNM – National Museum of Natural History, Washington D.C. (J. Coddington). ZMA – Zoologisch Museum Amsterdam (B. Brugge). ZMB – Museum für Naturkunde, Berlin (J. Dunlop, B. Nitsche). ZMH – Zoologisches Institut und Zoologisches Museum, Hamburg (H. Dastych). ZMUC – Zoological Museum of the University of Copenhagen (N. Scharff).

In the species descriptions the spider material is listed as follows: localities are listed from North to South, then from West to East; countries, provinces and towns/villages are listed as far as possible by their presently valid names.

## Results

### Characteristics of pre-epigynes

**Distinction of pre-epigyne from adult epigyne.** Pre-epigynes are considerably smaller than epigynes. If there is no adult female available to compare the size of the epigyne with that of the pre-epigyne of a subadult female the slit sense organs (SO) and epigynal muscle sigillae (EM) in front of the pre-epigyne can help. The distance between the SO from left to right side is about twice as long as the width of a pre-epigyne, but only slightly longer than the width of an adult epigyne. Furthermore, the pre-epigyne is only slightly longer than one EM. The adult epigyne, in contrast, is at least twice as long as EM. Moreover, the pre-epigyne exhibits either no epigynal field or the latter does not reach SO and/or EM.

**Ontogeny of the epigyne.** Pre-epigynes from four pseudo-orbweaverspecies were examined and found to exhibit apparently species-specific characteristics. Basic structures of adult epigynes can be recognised as primordial structures in the pre-epigynes. The following general ontogenetic process apparently leads from the primordial to the adult female copulatory organ: The anterior part of median septum (AS) and the anterior margins of lateral lobes (AML) extend strongly anterio-laterally.

In the subadult female of *Fecenia protensa* Thorell, 1891 the transverse ridge/edge of the median septum (TR) is clearly recognisable as a broad “W”-shaped edge (Fig. 69). In addition to the changes that happen from the subadult to adult stage described above, the median section of TR becomes strongly notched, together with a distinct median folding of AS. The result is the characteristic adult epigyne (Figs 55, 64, 108).

In *Fecenia cylindrata* Thorell, 1895, AML run at more or less a right angle anteriorly and face each other. This can be recognised overall in pre-subadult, subadult and adult females (Figs 79–82). A clearly developed TR is only present in subadult females and adults. In pre-subadult females the TR is at best only slightly indicated (dotted line in Fig. 82).

In *Fecenia ochracea* (Doleschall, 1859), it is easy to identify corresponding structures of subadult females and adults, because the pre-epigyne (Figs 20–21) already strongly resembles the adult one (Fig. 19). TR is present in subadult females. As on both sides TR is strongly curved anteriorly the characteristic broad-“nose-like” AS, like in adults (Fig. 19), is already recognisable. By contrast, in pre-subadult females TR is at best very weakly developed (Fig. 22).

In *Fecenia travancoria* Pocock, 1899, the situation is very similar to that in *Fecenia protensa*, although its pre-epigyne (Fig. 74) slightly differs from that of *Fecenia protensa* (Figs 58, 69) (see respective species descriptions).

**Different developmental stages of pre-epigynes.** Epigynes of adult females within the same species are similarly shaped (this is the reason why they can serve as an identification tool). In general this applies to the pre-epigynes, too. Yet, in one out of fifteen subadult females of *Fecenia cylindrata* the pre-epigyne was larger and somewhat differently shaped (Fig. 80) than generally (Figs 81, 94). It gives the impression that it may be further developed than the others. This phenomenon of a differing character state of the pre-epigyne does not mean that identification via the pre-epigyne is not possible. Because if the respective pre-epigyne is interpreted accurately, it is noticeable that it tends to fall along a developmental continuum together with the “regularly” shaped pre-epigynes, the pre-pre-epigynes of p.s.a. ♀♀ and the adult epigynes (Figs 79–82). The s.a. ♀ of *Fecenia cylindrata* illustrated in Fig. 80 is already more similar to the adult (Fig. 79). Its pre-vulva already exhibits a clear division into a transparent section of internal duct system (TSI) and a strongly sclerotised one (SSI) (Fig. 86). Hence, it is clearly recognised as *Fecenia cylindrata*.

In summary, pre-epigynes are easily distinguished from adult epigynes and apparently exhibit species-specific characters (note that one species pair *Fecenia protensa*/*Fecenia travancoria* is difficult to distinguish, but this is not surprising as it applies to the adults too; see respective species descriptions). In rare cases, in *Fecenia cylindrata* pre-epigynes of particular subadult females may differ from the general type. But by the means of an accurate interpretation of those pre-epigynes the respective subadult females can be recognised as *Fecenia cylindrata*, anyway. So, in *Fecenia* the pre-epigynes can be used as an identification tool. Here they are integrated in an identification key for the first time.

## Taxonomy

### Psechridae Simon, 1890

In combination, the following characters are diagnostic for Psechridae: cribellum and calamistrum present; claw tufts distally on the 3-clawed tarsi; rectangular calamistrum comprising at least 3 rows of setae; indirect eyes with grate shaped tapetum (Simon 1892, Homann 1950, Lehtinen 1967, Levi 1982, Griswold et al. 2005).

### Key to genera

**Table:** 

1	AME smaller than or as large as other eyes; opisthosoma ventrally mostly with white or beige median line; clypeus at least twice as high as diameter of AME; legs II and IV almost equal in length; build horizontal, dome-shaped sheet webs	*Psechrus*
–	AME larger than all other eyes; opisthosoma ventrally mostly with pair of white or beige patches, never with light median line; clypeus not or just slightly higher than diameter of AME; leg IV shorter than leg II; adults build vertical pseudo-orbwebs	*Fecenia*

#### Fecenia

Simon, 1887

http://species-id.net/wiki/Fecenia

Mezentia Thorell 1881: 203 (Type species: *Mezentia angustata* Thorell, 1881); Simon 1885: 451.
Fecenia Simon 1887: 194 (homonym recognised, *Mezentia* Stål, 1878 [Orthoptera], replacement name established); Simon 1890: 80; Simon 1892: 226; Lehtinen 1967: 234, 383 (syn. of type species *Fecenia angustata* with *Fecenia ochracea*); Levi 1982: 131; Coddington 1990: 7; Murphy and Murphy 2000: 264; Griswold et al. 2005: 38.

##### Diagnosis.

*Fecenia* species differ from *Psechrus* in the following characters: AME larger than all other eyes (in *Psechrus*, AME smaller or at most as large as other eyes); ventral side of opisthosoma centrally with pair of two white or beige patches, never with light median line like in *Psechrus*; clypeus flatter than in *Psechrus*, not or just slightly higher than diameter of AME, hence cephalic part of carapace rather flat; leg IV always shorter than leg II (in *Psechrus*, leg IV slightly longer or as long as leg II); in contrast to *Psechrus*, males with RTA, RPA, VPA and MA; females with clearly divided median septum of epigyne, vulva always lacking spherical spermathecal heads (in *Psechrus* females, median septum simple and spherical spermathecal heads generally present).

##### Description.

Medium sized to large Psechridae, body length in males: 7.2–13.2 mm; females: 7.7–20.2 mm. Cephalic part of carapace not distinctly narrower than broadest (thoracic) section. Anterior eye row recurved, posterior row straight (or at least almost straight). Chelicerae strong, shorter than in *Psechrus*, basal article at most 2.5 times longer than broad. Cheliceral furrow with three promarginal and four retromarginal teeth. Basal article of chelicerae ventrally with long field of short, transverse striae. Ventral surface of former distally with semicircular lobe with long, curved hairs (Fig. 6). Labium slightly longer than broad (Fig. 5). Gnathocoxae ca. twice as long as broad, distal section slightly broader than basal one (Fig. 5). Serrula with ca. 130–170 (size-dependant) very small, dark, apically blunt teeth, very densely arranged. Sternum slightly longer than broad, with pointed posterior ending and broad-angled (160°) anterior ending (Fig. 4). Pedipalp in females with single claw (Fig. 51) containing 8–12 teeth. Legs extremely long in males (metatarsus I ca. three times longer than carapace (Fig. 117), relatively long in females (metatarsus I ca. 1.5–2 times longer than carapace, Fig. 119). Leg formula 1243. Coxae of legs I, II broader than III, IV. Calamistrum dorso-retrolaterally on metatarsus IV consisting of 3–4 rows of setae (inner rows irregular). Spination of palp and legs: Highly variable within each species. Therefore, no species-specific and no common genus-specific spination pattern could be found. Consequently the spination will only be listed for the primary type specimen in the species descriptions. At the following positions spines are always absent: All patellae, dorsal surface of all tibiae and all metatarsi. Palpal femur spination varies from 000, 010, 110, 120, 130, which are the most common ones, to 141. Palpal patella, tibia and tarsus mostly without spines, if present, then very small, the most common patterns in this case are: patella 110, tibia 0100, tarsus 1004. Femora of legs I and II with even more variable spination, e.g. 100, 110, 210, 300, 310, 312, 320, 401, 412, 501, or 613. The most common one is 310. The same for those of legs III and IV, but here the number of spines is lower on average, most common is 010. The tibial spination pattern in *Fecenia* includes a characteristic aspect: Legs I and II: retrolateral spines absent; legs III and IV: prolateral ones absent. At each opposite side the number of spines varies from 0 to 4, with legs I and II mostly having one to two spines more than III and IV. Ventrally at tibiae I and II there are mostly 6, at tibiae III and IV mostly 4 spines (paired spines at all tibiae). The spination of metatarsi is more conservative: I–II 2015, III 1025 or 1015, IV 1015 (ventrally the four proximal spines are paired). But there are exceptions, too. Colouration: Chelicerae, carapace and sternum yellowish brown to dark brown. In rare cases specimens exhibiting a darker carapace margin and a median longitudinal band. Sternum unicoloured. Legs from yellowish brown or light brown to brown, may be annulated. Tibiae I and II in some cases darker than other limbs/legs. Femora at distal third often with dark, annulated patches. Opisthosoma dorsally greyish-brown with yellowish patches. Heart region with darker lanceolate patch with light centre (Fig. 119). Distal half of opisthosoma dorsally with two converging rows of dark brown spots. Lateral surface of opisthosoma is covered with 3–4 larger yellowish patches running diagonally. Opisthosoma ventrally dark brown to black, centrally with a pair of white to beige patches (Figs 116, 118), which differ intraspecifically in size and shape. In some cases those patches are fused, in extremely rare cases absent. Additionally, with white to beige transverse patch in front of spinnerets/cribellum (Fig. 116). The whole body is covered with grey hairs (Fig. 116). Spinnerets are relatively short and conical, except for median ones, which are distinctly smaller, slender and cylindrical. Bipartite character clearly visible in posterior spinnerets. Copulatory organs: Male palp with almost round tegulum (T). Embolus (E) filiform, arising in prolateral half of tegulum (T) and at least twice as long as conductor (C). The latter membranous, mostly arising centrally on upper half of T (Fig. 8) and mostly shorter than median apophysis (MA). T next to E-base (Fig. 8) with membranous process (MP). MA relatively large with general retrolateral direction (e.g. Fig. 89). Cymbium distinctly broader than palpal tibia and patella (e.g. Fig. 62). RTA differently shaped among the particular species, DRTA only present in *Fecenia macilenta* (Simon, 1885) (Figs 53–54). VPA often slightly bent anteriorly (e.g. Fig. 87). RPA mostly small and inconspicuous. Palpal femur modifications, e.g. ventral bulge as present in some *Psechrus* species, absent in all *Fecenia* species. Scopula dorsally on cymbium present in the same form in all *Fecenia* species (Figs 99–101), but less distinct than in most *Psechrus* species. Female epigyne generally broader than long, with folded median septum (e.g. Fig. 55). Anterior part of median septum (AS) larger than posterior part (PS). Anterior margins of lateral lobes (AML) iin some species strongly sclerotised (Fig. 108). Vulva simple, with internal duct system divided in three sections: Transversal section (TSI), strongly sclerotised section (SSI) and fertilisation duct (FD) (Fig. 83). Borderline (BL) between TSI and SSI clearly recognisable and often of taxonomic importance.

##### Biological notes.

The pseudo-orbweavers are found in shrubs and trees, and also in the canopy (Deeleman, pers. comm.). *Fecenia* suspends its vertical pseudo-orbweb (Fig. 120) in the vegetation, mostly between twigs. The web possesses an enrolled leaf at the hub serving as a retreat. This is true for adults and later instar juveniles of all *Fecenia* species. Earlier instars build an elongate cone-shaped tube as a retreat, which is disguised with small prey remains and soil- and leaf-particles. The very early instars do not even build a pseudo-orbweb, but a rather conical or dome-shaped web with the retreat at the top of the cone. This web can be found in the herb layer too (Robinson and Lubin 1979). The pseudo-orbweb (Fig. 120) is more irregular than the webs of araneids and related families building orb-webs. In *Fecenia* there is no regular spiral of capturing thread(s) as in araneids etc. In *Fecenia*, one cannot speak of a real spiral as the distance between the threads and their orientation differs. The irregularity applies to the radii too. In many cases they are not continuous.

Predatory behaviour was observed in the lab using several *Fecenia cylindrata* and *Fecenia protensa* specimens. In each case the spider was transferred to a large cylindrical glass (30 cm high, diameter 20 cm) with a leaf, already partly enrolled, placed at the bottom. The next day the leaf was suspended by threads in the middle of the glass, a day later it was already fixed at the top. The pseudo-orbweb was completed another day later. After placing a house fly into the lower area of the web it took a few seconds until the spider stretched its two forelegs out of the retreat, and after ca. 1 minute it came out. The fly was grabbed with the chelicerae and immediately dragged into the retreat. A few centimetres before the leaf entrance the spider turned and crawled backwards into the retreat. In the case of larger prey items like crickets, the spider was extremely shy and careful. It took two or three attempts of coming out of the leaf and escaping back into it, sometimes interrupted by 5–15 minutes within the retreat. During the last attempt the cricket was bitten for about 7 minutes at the capturing site of the web before it was dragged to the retreat. Binding behaviour, as described in Robinson and Lubin (1979), was observed after providing an even larger cricket. But in addition to their observations I could recognise that *Fecenia* took threads out of the web, too, in order to bind its prey. An attack-wrapping behaviour like in Araneidae does not exist.

Robinson and Lubin (1979) observed the mating behaviour of a male *Fecenia ochracea* (however in their publication identified as *Fecenia* sp.) approaching the female retreat, which I corroborate observing (raised) *Fecenia cylindrata* from Champasak Province, Laos (males SB 509, 510 and females SB 486–487, 511, 514, see list in description of *Fecenia cylindrata*, additional material examined). These were maintained in cylindrical glasses (see above) and fed on house flies and crickets. A few days after one male's final moult its web was reduced to a few frame threads. In two corners of that thread-framework sperm webs were found (Fig. 123). The bulb filling procedure was not observed. A female was transferred into a terrarium (30 cm high, diameter 20 cm) and offered a small “cone” of transparent film as retreat, which was accepted and later on integrated in the new web. In the respective trial the male was placed into the female's terrarium. After a while it approached the retreat from the top of the terrarium by roping down onto it. There it tapped and stroked the retreat carefully (Fig. 121). Later on it moved to the margin of the opening of the leaf retreat and repeated this behaviour. After some more repeats it stayed there motionless. Unfortunately, neither the moment of entering the retreat nor the copulation itself could be observed. The next day the male was sitting within the leaf retreat, together with the female (Fig. 122). In another trial a raised *Fecenia protensa* male from Flores (SB 196, see description of *Fecenia protensa*, list of additional material examined) was transferred to a terrarium with an already adult conspecific female from Bali. The behaviour was the same as described above, but in this case the next day saw the male half digested lying at the bottom underneath the retreat, which means it had been attacked and killed by the female. In one further trial a *Fecenia protensa* male was put into the terrarium of a *Fecenia cylindrata* female*.* The approaching behaviour upon the leaf was executed up to the point when the male reached the leaf opening. Here he turned and disappeared to an upper corner of the terrarium and stayed there motionless for more than one day.

### Key to species:

**Table:** 

1	Male (that of *Fecenia travancoria* unknown)	2
–	Female (subadult one of *Fecenia macilenta* unknown)	5
2	DRTA absent, MA prominent, in some species massive	3
–	Prominent DRTA (Figs 53–54) present, MA slender and rather inconspicuous (Fig. 53)	*Fecenia macilenta*
3	RTA short, at most ½ the width of palpal tibia, MA shorter than width of T	4
–	RTA at least as long as width of palpal tibia, MA large and massive, at least as long as width of T (Fig. 10)	*Fecenia ochracea*
4	RTA knobbed, almost as broad as long, E without bEA, VPA arising proximally on patella (Figs 60–61)	*Fecenia protensa*
–	RTA rather slender, longer than broad, E with distinct, pointed bEA (Fig. 89), VPA arising centrally (Figs 87–88)	*Fecenia cylindrata*
5	AML distinctly visible, AS with similar colour as surrounding parts of epigyne	6
–	AML hardly visible, posterior half of AS distinctly darker than surrounding parts of epigyne (Figs 48, 114)	*Fecenia macilenta*
6	AML converging anteriorly and surrounding epigynal pit partly, pre-epigyne with TR running completely from left to right AML, in pre-vulva distance between centres of pre-receptacula > 3 x diameter of one pre-receptaculum	7
–	AML diverging anteriorly (Fig. 1), in pre-epigyne gaps between TR and AML (Figs 20–21), in pre-vulva distance between centres of pre-receptacula < 3 x diameter of one pre-receptaculum (Figs 23–24)	*Fecenia ochracea*
7	AS with longitudinal folding, the latter mostly anteriorly pointed (e.g. Figs 55, 76), TR with distinct notch, pre-epigyne with double curved TR, the latter broadly W-shaped (Fig. 69), in general appearance pre-epigyne looking crown-shaped, pre-receptaculum bulbous/spherical (Figs 67–68)	8
–	AS flat (at least anteriorly), without longitudinal folding (Fig. 79), TR without notch, pre-epigyne with continuous TR (Fig. 81), the latter slightly curved, pre-receptaculum with lateral extension (Fig. 85)	*Fecenia cylindrata*
8	In vulva BL running almost longitudinal (Fig. 77), lateral prongs of the “crown” in pre-epigyne narrow (Fig. 74)	*Fecenia travancoria*
–	In vulva BL running +/- transversal (Fig. 56), lateral prongs of the “crown” in pre-epigyne not distinctly narrow (Figs 58, 69)	*Fecenia protensa*


#### Fecenia ochracea

(Doleschall, 1859)

http://species-id.net/wiki/Fecenia_ochracea

Figs 1 [Fig F2] [Fig F3] [Fig F4] [Fig F5] [Fig F6] [Fig F7] [Fig F8] [Fig F9] –47 96 102–104 118

Tegenaria ochracea Doleschall, 1859: 50, pl. 8, fig. 8 (Description of ♀), [Holotype ♀ (SB 94) from INDONESIA: Maluku Prov.: Ambon Isl.; C. L. Doleschall leg. 1855–1858; NHMW 12∙389, examined]; Thorell 1878: 302 (sub *Tegenaria* [?]); Thorell 1881: 694 (sub *Tegenaria* [?]).
Mezentia angustata Thorell, 1881: 204 (Description of ♀), [Holotype ♀ (SB 460) from INDONESIA: Maluku Utara Prov.: Ternate Isl. next to Halmahera; Prof. O. Beccari leg. 1872–1877; MCSN, examined]; Simon 1885: 451.
Mezentia ochracea - Simon 1885: 451 (Transfer from *Tegenaria*).
Fecenia ochracea - Simon 1887: 194 (Formal transfer from *Mezentia*, preoccupied by Stål, 1878 in Orthoptera, replacement name *Fecenia*); Simon 1892: 226; Simon 1906: 287, fig. 1B (Illustration of ♀); Kulczyński 1908: 570; Reimoser 1936: 407; Lehtinen 1967: 234; Levi 1982: 133, figs 68–79, 90 (Illustration of ♂ and ♀♀); Murphy 1986: 65; Griswold 1993: 7; Murphy and Murphy 2000: plate 21, fig. 5 (Photo of ♀); Song et al. 2002: 373 (listed as fauna element of Singapore; doubtful!, to date no records from Singapore).
Fecenia angustata - Simon 1887: 194 (Formal transfer from *Mezentia*); Simon 1892: 226; Pocock 1900: 212; Kulczyński 1908: 570; Petrunkevitch 1928: 90; Reimoser 1936: 407; Chrysanthus 1967: 102, figs 55–57, 60–64 (Description of ♂, illustration of ♂ and ♀♀); Lehtinen 1967: 234 (Synonymy).
Fecenia maforensis Simon, 1906: 287, fig. 1A (Description of ♀), [Holotype ♀ (SB 464) from INDONESIA: Irian Jaya Barat Prov.: Numfor Isl., formerly Mafor; A. Raffray leg.; MNHN AR185, examined]; Kulczyński 1908: 570; Strand 1915: 191 (Description of ♀); Reimoser 1936: 407; Chrysanthus 1967: 104, fig. 65 (Illustration of ♀); Lehtinen 1967: 234 (Synonymy).
Fecenia montana Kulczyński, 1910: 389, pl. 17, fig. 1 (Description of ♀), [Holotype ♀ (SB 461) from PAPUA NEW GUINEA: East New Britain Prov.: Baining Mountains; K. Rechinger leg. 1906; NHMW 12∙388, examined], Reimoser 1936: 407, Lehtinen 1967: 234 (Synonymy).
Fecenia oblonga Rainbow, 1913: 7, fig. 5 (Description of ♀), [Holotype ♀ from SOLOMON ISLANDS: Western Prov., Shortland Island Group, Island of Howla; W. W. Froggatt leg. ca. 1900; AMS, lost (Milledge, AMS, pers. comm.), thus not examined]; Reimoser 1936: 407; Lehtinen 1967: 234 (Synonymy).
Fecenia cinerea Hogg, 1914: 56 (Description of ♀), [Holotype ♀ (SB 404) from INDONESIA: Papua Prov.: Possibly near Mount Utakwa; A.F.R. Wollaston leg. 1912–1913 (Wollaston Expedition in Dutch New Guinea); NHM 1921·3·24·9, examined]; Hogg 1915: 437, fig. 23 (Illustration of ♀); Reimoser 1936: 407; Lehtinen 1967: 234 (Synonymy).
Fecenia buruana Reimoser, 1936: 406, fig. 1 (Description of ♂ ♀), [Lectotype ♀ (SB 418), paralectotype ♂ (SB 417) by designation of Levi (1982: 134), both from INDONESIA: Maluku Prov.: Buru Isl., station 1; L.J. Toxopeus leg. 1921; ZMA, examined]; Chrysanthus 1967: 104, figs 66–67 (Illustration of ♂ and ♀); Lehtinen 1967: 234 (Synonymy).

##### Note on the holotype of *Tegenaria ochracea*.

The first description of Doleschall (1859) lacks any remarks concerning deposition of the type specimen. Generally, material recorded by naturalists of the “Natuurkundige Vereeniging in Nederlandsch Indie” has been deposited either in RMNH or in ZMA. Lehtinen (1967) stated that the type deposition was unknown (to him). Levi (1982) mentioned a personal communication from Van der Hammen, the curator of the arachnid collection in RMNH at that time, who stated that the type was lost. At present, the colleagues of the arachnid collection of RMNH still cannot find any type material of *Tegenaria ochracea* there (K. van Dorp and J. Miller, RMNH, pers. comm.). In the arachnid collection of ZMA there is also no type specimen of *Tegenaria ochracea* (B. Brugge, pers. comm.). During a stay at the natural history museum in Vienna in April 2009 I recognised a syntype specimen of *Psechrus argentatus* (Doleschall, 1857). Both Lehtinen (1967) and Levi (1982) believed that the syntypes of this species had been lost. However, for this latter species found on Ambon, too, and recorded and described by the same author just two years before, it is evident that at least a part of the original syntype series was once deposited in RMNH (Van Hasselt 1877). After consulting Jürgen Gruber and Verena Stagl (both NHMW) I learned that Doleschall sent only a part of his spider- and insect material collected on Ambon to the museum in Leiden; a large part of the material was sent to the museum in Vienna (Stagl 1999). In the spider collection of NHMW I found a *Fecenia* female (SB 94), which was labelled “*Fecenia* - Insel Ambon” (oldest label). According to Gruber (pers. comm.) the handwriting is that of E. Reimoser, the curator of NHMW from 1923–1940. It is well known that Reimoser often discarded old labels and substituted them with new ones (Gruber pers. comm.). It is most likely that in this case the same had happened. Assuming that the handwriting on the original label from Doleschall was unclear, it is likely that Reimoser discarded that label, determined the female as *Fecenia* and just added the locality on the new label. Anyway, it is evident that before 1950 nobody other than Doleschall sent spider material from the island Ambon to the natural history museum in Vienna (Gruber pers. comm.). Hence, the female SB 94 (see synonymy list above) can be considered the holotype of *Tegenaria ochracea*.

##### Additional material examined.

(4 ♂♂, 73 ♀♀, 4 s.a. ♂♂, 7 s.a. ♀♀, 2 p.s.a. ♀♀, 11 juvenile specimens)**. PHILIPPINES:** Luzon: Laguna Prov.: Los Baños; Baker leg.; 1 ♀ (SB 153), MCZ 82529. **MALAYSIA:** **Borneo: Sabah Prov.:** Kinabalu N.P., Poring Hot Springs, 5°02'N, 116°42'E, 600–700 m, primary forest; A. Floren leg. 03.III.1996 by canopy fogging “ridge”; 1 ♀ (SB 518), Deeleman Coll. in RMNH. **INDONESIA:** **Sumatra: Nanggroe Aceh Darussalam Prov.:** Ketambe, Gunung Leuser N.P., 3°51'N, 97°37'E, ca. 1300 m, primary forest, from leaves; S. Djojosudharmo leg. 03.V.1986; 1 ♀ (SB 127), Deeleman Coll. in RMNH. **Halmahera: Maluku Utara Prov.:** Jailolo Distr., Kampung Pasir Putih, 0°53'N, 127°41'E; A.C. Messer, P.M. Taylor leg. 1981; 1 ♂ (SB 187), USNM. **Maluku Utara Prov.:** Ternate Isl.; A. Raffray leg.; 1 s.a. ♂ (SB 465), MNHN. **Maluku Prov.:** Buru Isl., station 1; L. J. Toxopeus leg. 1921; 1 ♀ (SB 419), ZMA. Ceram Isl.; 6 ♀♀ (SB 470–473, 475–476), 1 s.a. ♂ (SB 467), 1 s.a. ♀ (SB 469), 1 juv. (SB 468), MNHN AR193. Ambon Isl.; 1 ♀ (SB 474), MNHN AR193. Aru Isls; 1 s.a. ♀ (SB 80), Roewer Coll. 1819 in SMF. **Irian Jaya Barat Prov.:** Manokwari, Dorey; A. Raffray leg.; 1 ♀ (SB 466), MNHN. **Papua Prov.:** Sentani; leg. IV. 1903; 1 ♀ (SB 661), MIZ. Mindiptana; B. Monulf leg. 1958-1965; 3 ♀♀ (SB 96-98) Coll.-No. 8474, 1 ♂ (SB 95), 1 ♀ (SB 442) Coll.-No. 8476, all RMNH. Merauke; B. Monulf leg. 1956-1957; 16 ♀♀ (SB 426-441) Coll.-No. 8475, 8 ♀♀ (SB 443-447, 450, 452-453), 1 s.a. ♂ (SB 444) Coll.-No. 8477, 4 ♀♀ (SB 99-102) Coll.-No. 8478, all RMNH. **Java: Jawa Barat Prov.:** Gunung Gedeh N.P., Cibodas Nature Reserve, 6°44'S, 107°00'E, 1450 m; S. Djojosudharmo leg. 06.XII.1986; 1 ♂ (SB 120), Deeleman Coll. in RMNH. **PAPUA NEW GUINEA:** **West Sepik Prov.:** Aitape, Seleo; 1 ♀ (SB 662), MIZ. **Morobe Prov.:** Wau, 7°20'S, 146°43'E; M. Robinson leg. 10–15.IV.1977, 5 ♀♀ (SB 163–166, 484), 2 juvs (SB 482–483); H. Levi, Y. Lubin, M. Robinson leg. 07.–12.III.1979, MCZ 82521, 5 ♀♀ (SB 156–157, 162, 479–480), 1 s.a. ♀ (SB158), MCZ 82533, J.E. Carico leg. 22.–29.VI.1982, 2 ♀♀ (SB 154–155), 1 p.s.a. ♀ (SB 478), 1 juv. (SB 477), MCZ 82531. Wau; 7°20'S, 146°43'E; J.E. Carico leg. 05.–06.VII.1982; 1 ♂ (SB 180), USNM. Wau, Ecology Center; E.I. Schlinger leg. 17.II.1978; ♀ (SB 947), CAS 9032225. **East New Britain Prov.:** “Putie Bucht”, South coast; Dr G. Ducker leg. 05.-19.II.1909, Hamburg Südsee Exp., No. 300; 1 s.a. ♀ (SB 896), ZMH. Jacquinot Bay, ca. 5°34'S, 151°26'E; Dr G. Duncker leg. 19.-20.XII.1908, Hamburg Südsee Exp., No. 261; 2 ♀♀ (SB 892-893), ZMH. Keravat, 4°21'S, 152°07'E, 300 m, lowland tropical rain forest; I. Agnarsson leg. 03.–07.IV.2009; 1 s.a. ♀ (SB 540), 1 juv. (SB 541), SMF. Keravat, Laes; Y.D. Lubin leg. 01.VII.1980; 1 ♀ (SB 167), MCZ 82525. Kokopo, Ralum, ca. 4°20'S, 152°15'E, ca. 50 m; F. Dahl leg. 12.X.1896; 1 ♀ (SB 801), 1 s.a. ♀ (SB 794), 4 juvs (SB 795–800), ZMB 15472, 19244–19248. “Dörper Spitze, S.O. Bucht”: Dr G. Duncker leg. 14.V.1909, Hamburg Südsee Exp., No. 534; 2 ♀♀ (SB 894-895), ZMH. **New Ireland Prov.:** New Ireland, Lemkamin; Nocna Dan Exp. 1961-1962; 1 ♀ (SB 887), ZMUC 5728. Feni Isls, Ambitle Isl. (Anir); E. Wolf leg. 04.V.1909; 1 ♀ (SB 86), SMF 2769/1. **Papua New Guinea [no other locality data]:** L. Biro leg.; 1 ♀ (SB 668), 1 p.s.a. ♀ (SB 669), 2 juvs (SB 670-671), MIZ 46/51U. **SOLOMON ISLANDS:** New Georgia Group; J.F. P. leg. 1965; 1 s.a. ♀ (SB 392), NHM. Auki; W.M. Mann leg. 1916; 3 ♀♀ (SB 159–161), MCZ 82524.

##### Diagnosis.

**Figures 1–6.**
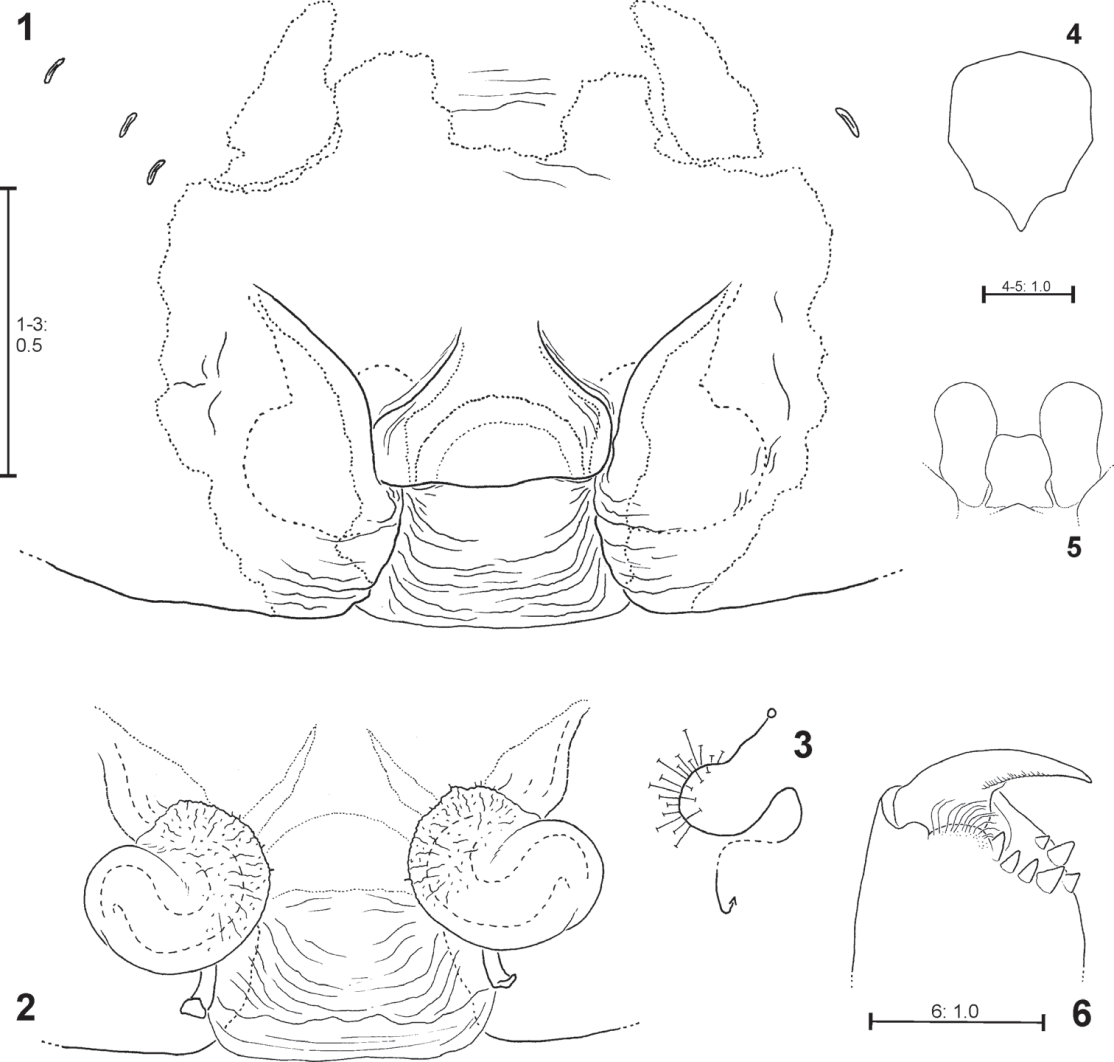
*Fecenia ochracea*, ♀ holotype (SB 94) from Ambon, Indonesia **1** Epigyne, ventral view **2** Vulva, dorsal view **3** Schematic course of internal duct system, dorsal view **4** Sternum, ventral view **5** Labium and gnathocoxae, ventral view. **6** Right chelicere, ventral view.

Distinguished from other *Fecenia* species by the epigyne with diverging anterior margins of lateral lobes (AML) (Fig. 1). Males differ from all other *Fecenia* species by RTA at least as long as width of palpal tibia, MA large and massive, at least as long as width of tegulum (T) (Fig. 8).

##### Description.

**Figures 7–10. F2:**
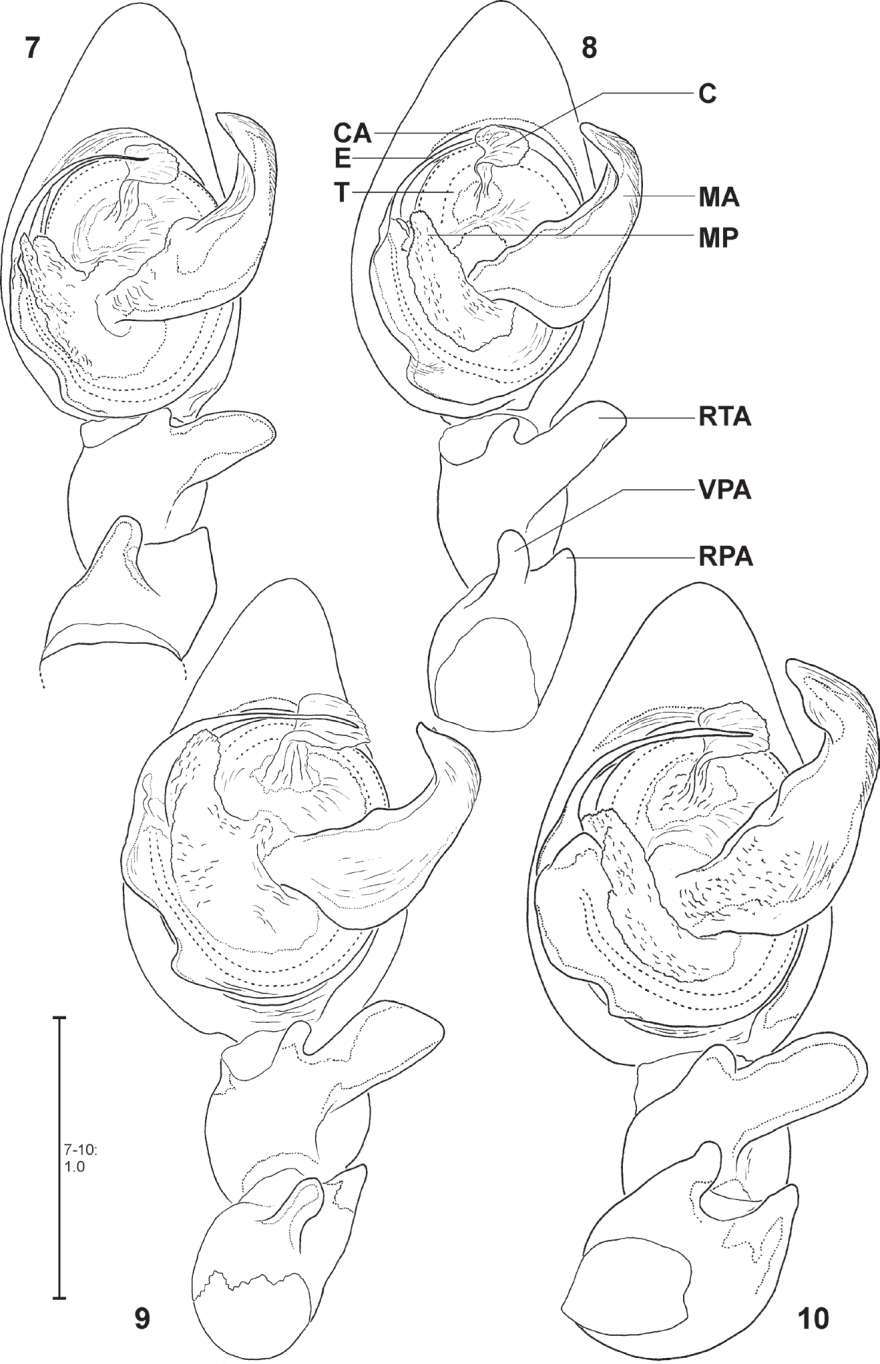
*Fecenia ochracea*, left ♂ palp, ventral view **7** SB 120 from Java, Indonesia **8** Paralectotype of *Fecenia buruana* (SB 417) from Buru, Indonesia **9** SB 95 from Mindiptana, Indonesia **10** SB 180 from Wau, Papua New Guinea. Remark on Fig. 8 Embolus slipped behind conductor. C = Conductor; CA = Cymbium alveolus; E = Embolus; MA = Median apophysis; MP = Membranous process of tegulum; RPA = Retrolateral patellar apophysis; RTA = Retrolateral tibial apophysis; T = Tegulum; VPA = Ventral patellar apophysis.

**Figures 11–14. F3:**
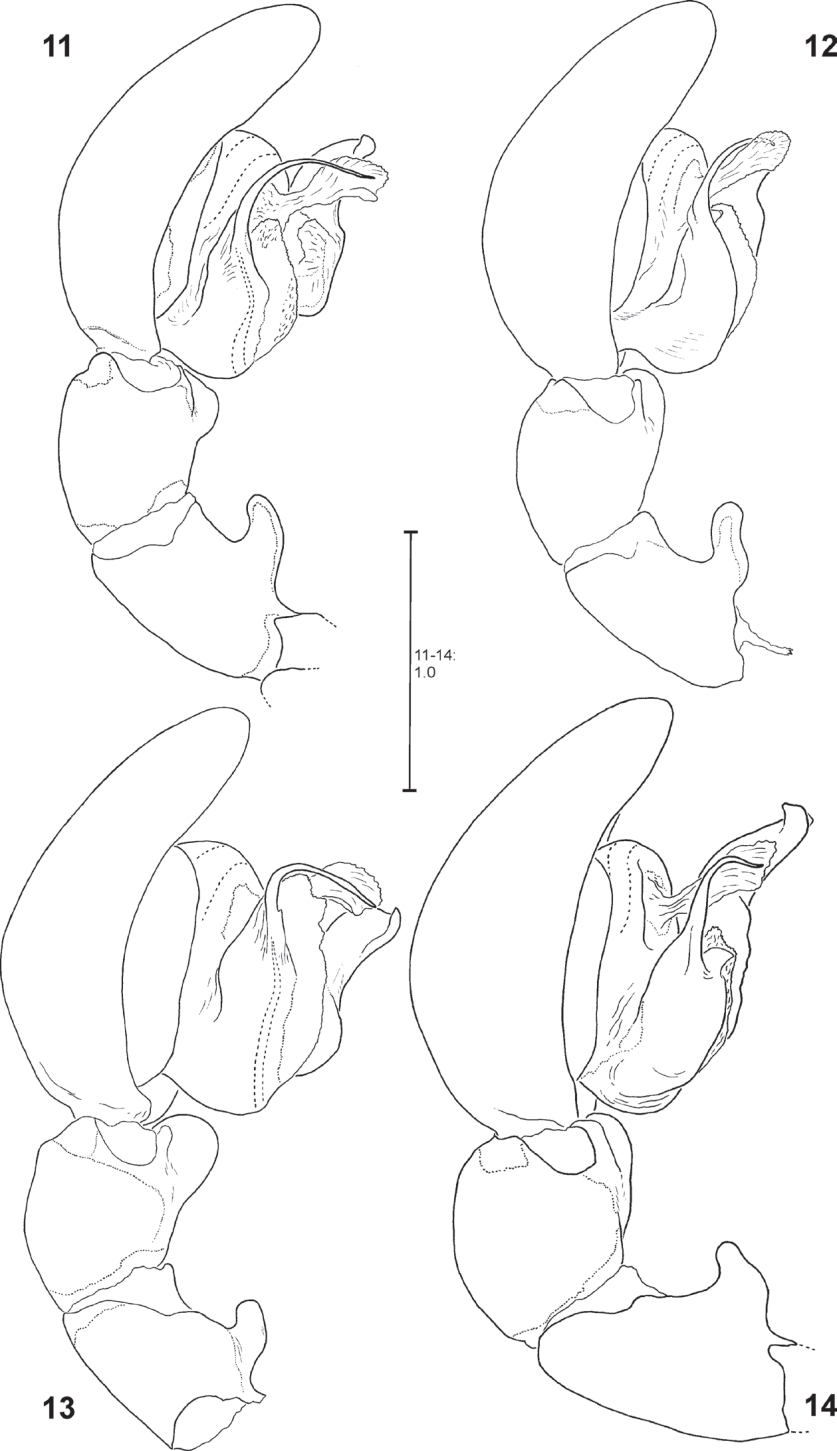
*Fecenia ochracea*, left ♂ palp, prolateral view **11** SB 120 from Java, Indonesia **12** Paralectotype of *Fecenia buruana* (SB 417) from Buru, Indonesia **13** SB 95 from Mindiptana, Indonesia **14** SB 180 from Wau, Papua New Guinea.

**Figures 15–18. F4:**
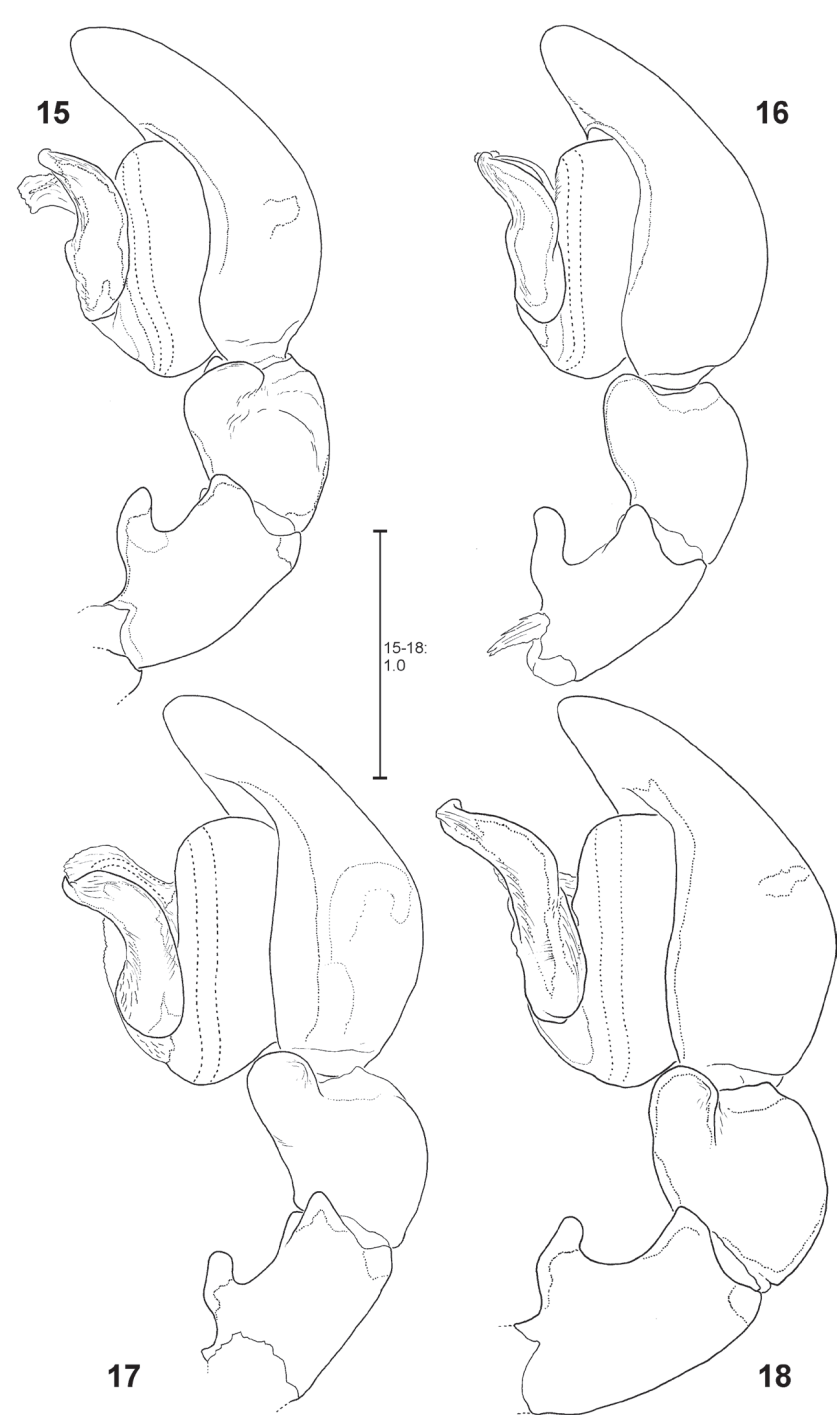
*Fecenia ochracea*, left ♂ palp, retrolateral view **15** SB 120 from Java, Indonesia **16** Paralectotype of *Fecenia buruana* (SB 417) from Buru, Indonesia. **17** SB 95 from Mindiptana, Indonesia **18** SB 180 from Wau, Papua New Guinea.

MALE:Body and eye measurements. Carapace length 4.2–4.7, carapace width 2.8–3.4, anterior width of carapace 1.7–2.1, opisthosoma length 4.8–7.1, opisthosoma width 2.0–3.3. Eyes: AME 0.28–0.33, ALE 0.20–0.23, PME 0.20–0.23, PLE 0.20–0.22, AME–AME 0.17–0.28, AME–ALE 0.06–0.13, PME–PME 0.22–0.28, PME–PLE 0.28–0.42, AME–PME 0.14–0.17, ALE–PLE 0.11–0.17, clypeus height at AME 0.28–0.42, at ALE 0.21–0.34. Measurements of palp and legs. Palp 5.2–6.1 [2.0–2.4, 0.8–1.1, 0.7–0.8, 1.3–1.8], I 46.6–55.9 [12.6–15.6, 1.9–2.2, 12.3–15.5, 13.4–16.9, 5.2–5.7], II 21.7–26.8 [5.8–6.7, 1.5–1.8, 6.0–7.6, 6.0–7.0, 2.4–3.0], III 12.1–14.2 [3.4–4.1, 1.1–1.4, 3.0–3.6, 3.1, 1.4–1.7], IV 20.6–24.0 [5.4–6.8, 1.4, 5.4–6.5, 6.2–6.5, 2.2–2.6]. Leg formula: 1243. Copulatory organ: Ventral patellar apophysis (VPA) arising in basal third of palpal patella (Figs 11–18), retrolateral patellar apophysis (RPA) mostly inconspicuous (Figs 9, 17). RTA distally not or just slightly broader than basally (Fig. 10). MA ventrally in basal third with distinct bulge (Figs 8–9, 96). Distal part of MA bent prolaterally. General direction of MA 1:00 or 1:30-o'clock. Embolus (E) arising in ca. 9-o'clock-position on T, at most as long as width of T (Figs 7–10, 96). T with corner-like lobe ventrally in prolateral half, T slightly longer than broad. MP with differing lengths (Figs 7–10, 96). Conductor (C) small, arising centrally in upper third of T.

**Figures 19–26. F5:**
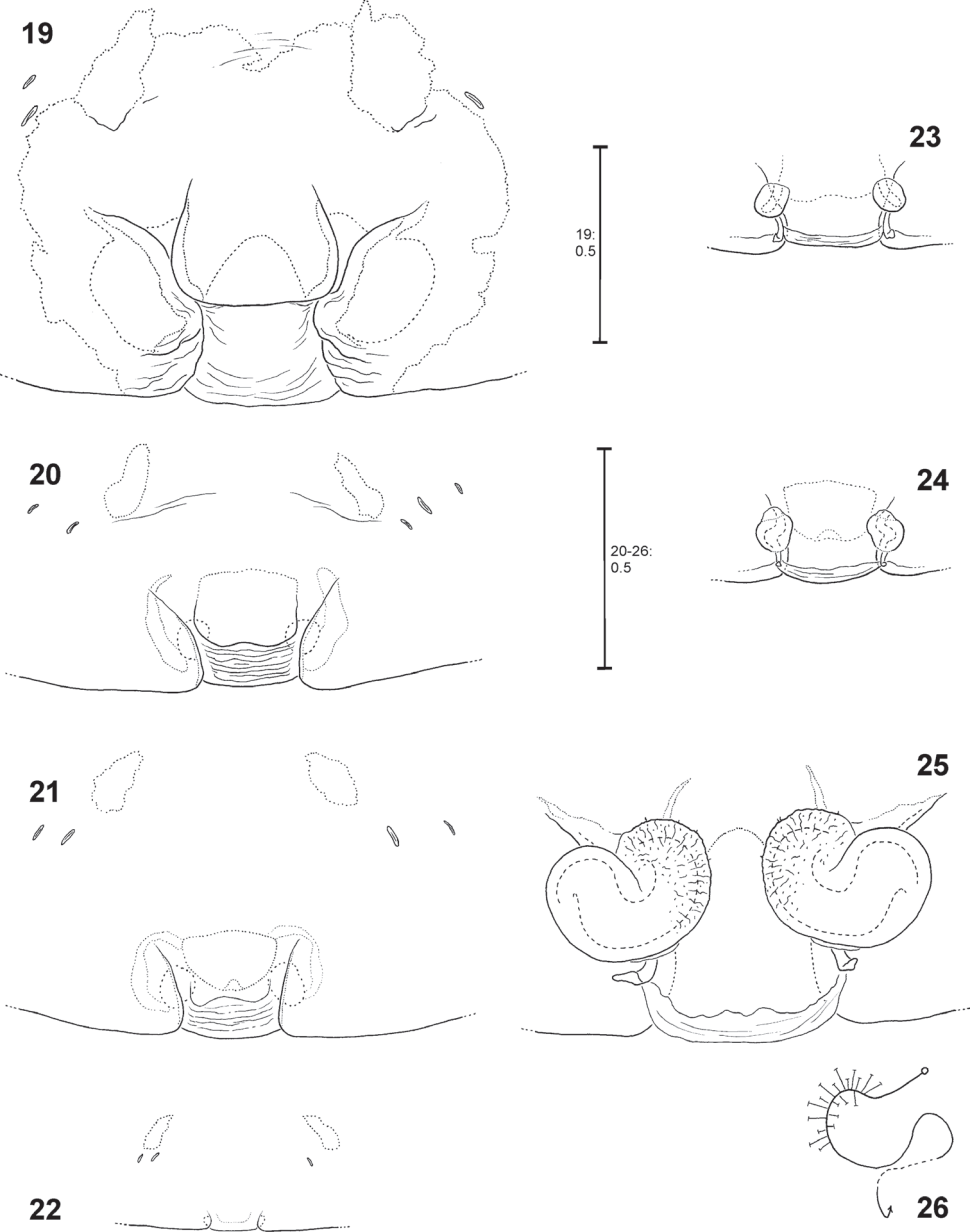
*Fecenia ochracea*, ♀ copulatory organ/primordial copulatory organ **19, 25–26** Holotype ♀ of *Fecenia montana* (SB 461) from East New Britain **20, 23** s.a. ♀ SB 540 from East New Britain **21, 24** s.a. ♀ SB 158 from Wau, Papua New Guinea **22** p.s.a. ♀ SB 669 from New Guinea **19** Epigyne, ventral view **20–21** Pre-epigyne, ventral view **22** Pre-pre-epigyne, ventral view **23–24** Pre-vulva, dorsal view **25** Vulva, dorsal view **26** Course of internal duct system.

**Figures 27–28. F6:**
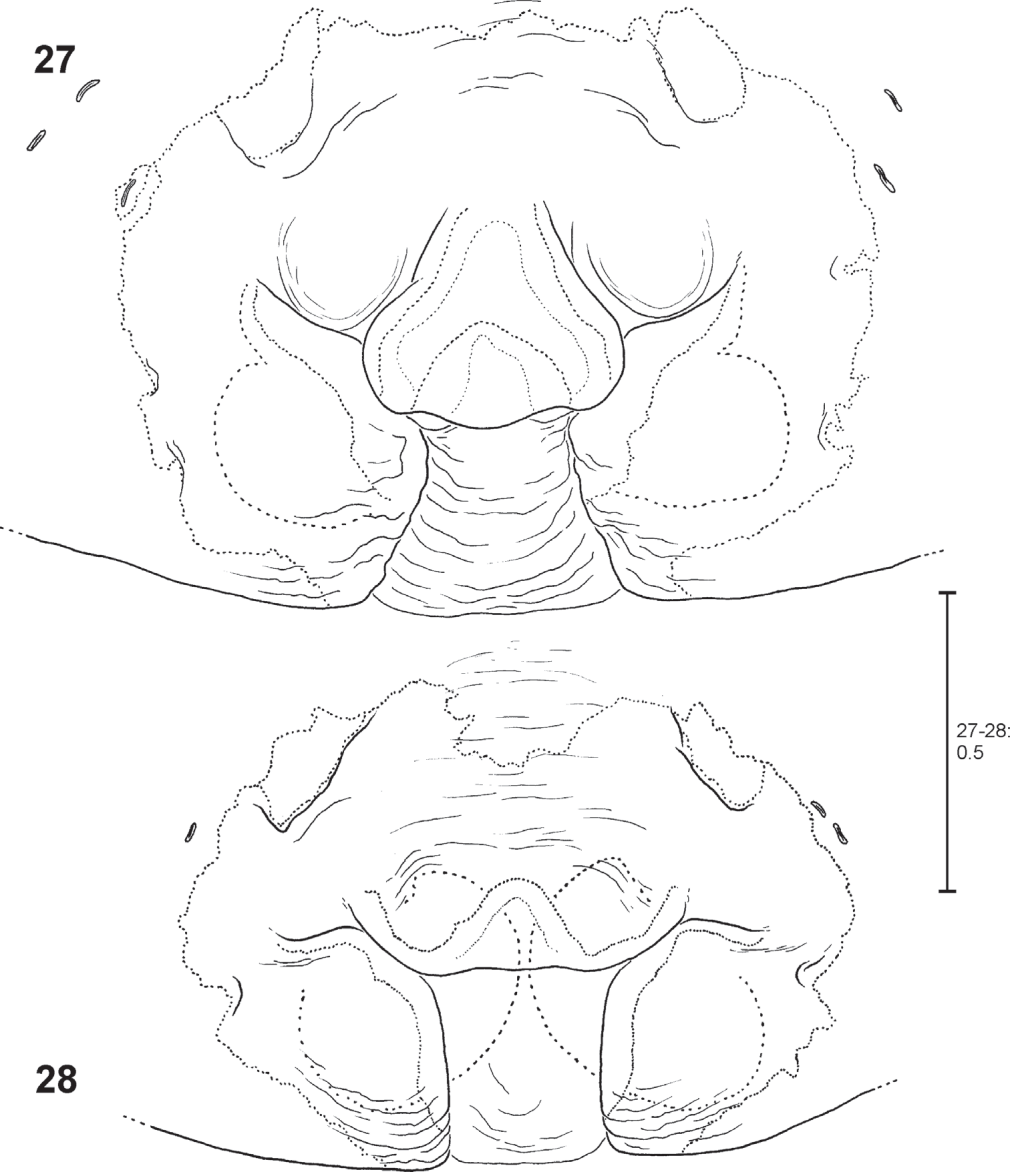
*Fecenia ochracea*, ♀ epigyne, ventral view **27** Holotype of *Fecenia maforensis* (SB 464) from Northwestern Irian Jaya, Indonesia **28** Holotype of *Fecenia cinerea* (SB 404) from Southern Papua Prov., Indonesia.

**Figures 29-30. F7:**
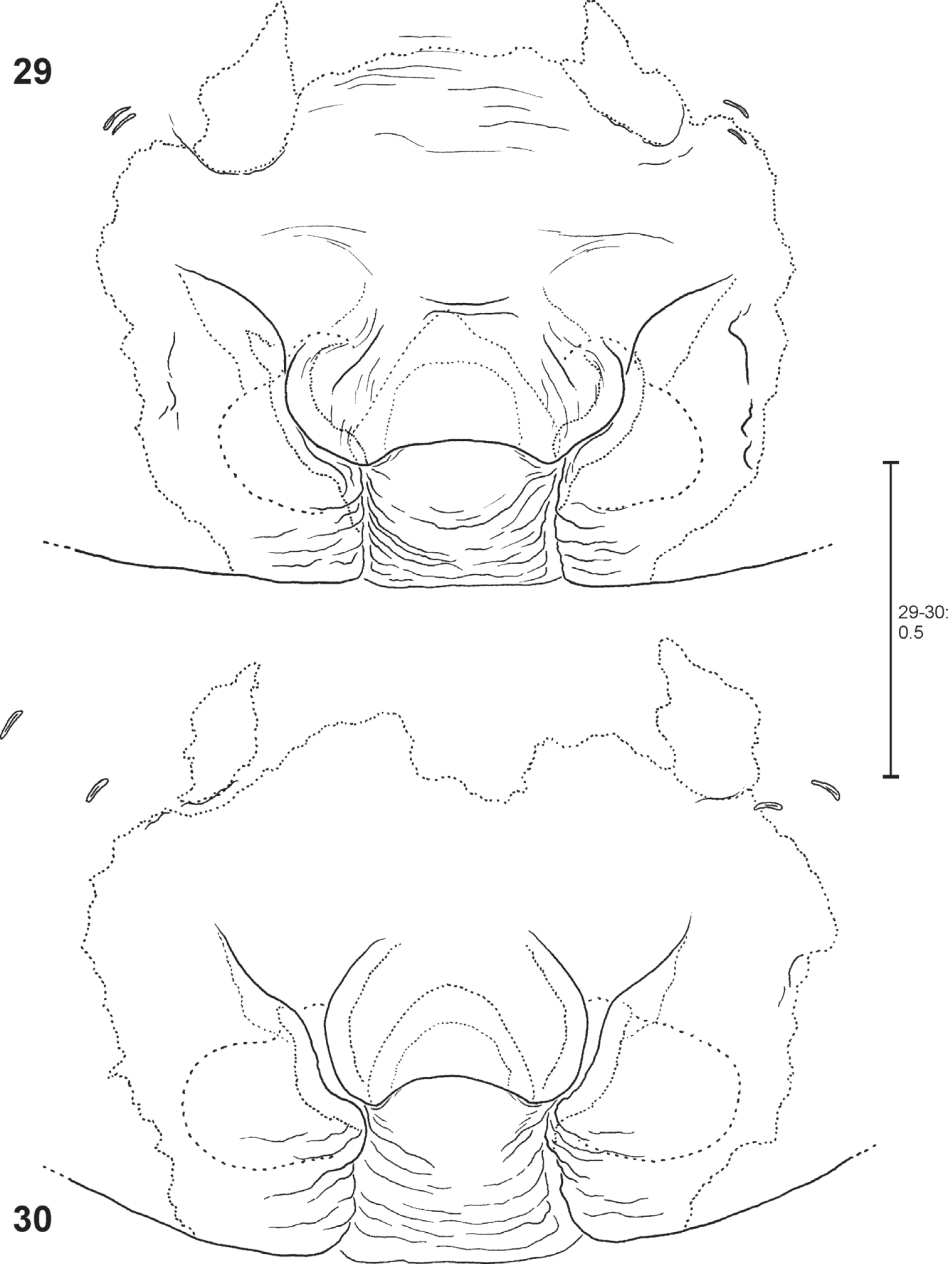
*Fecenia* ochracea, ♀ epigyne, ventral view **29** Holotype of *Fecenia angustata* (SB 460) from Ternate, Indonesia **30** Lectotype of *Fecenia buruana* (SB 418) from Buru, Indonesia.

**Figures 31–33. F8:**
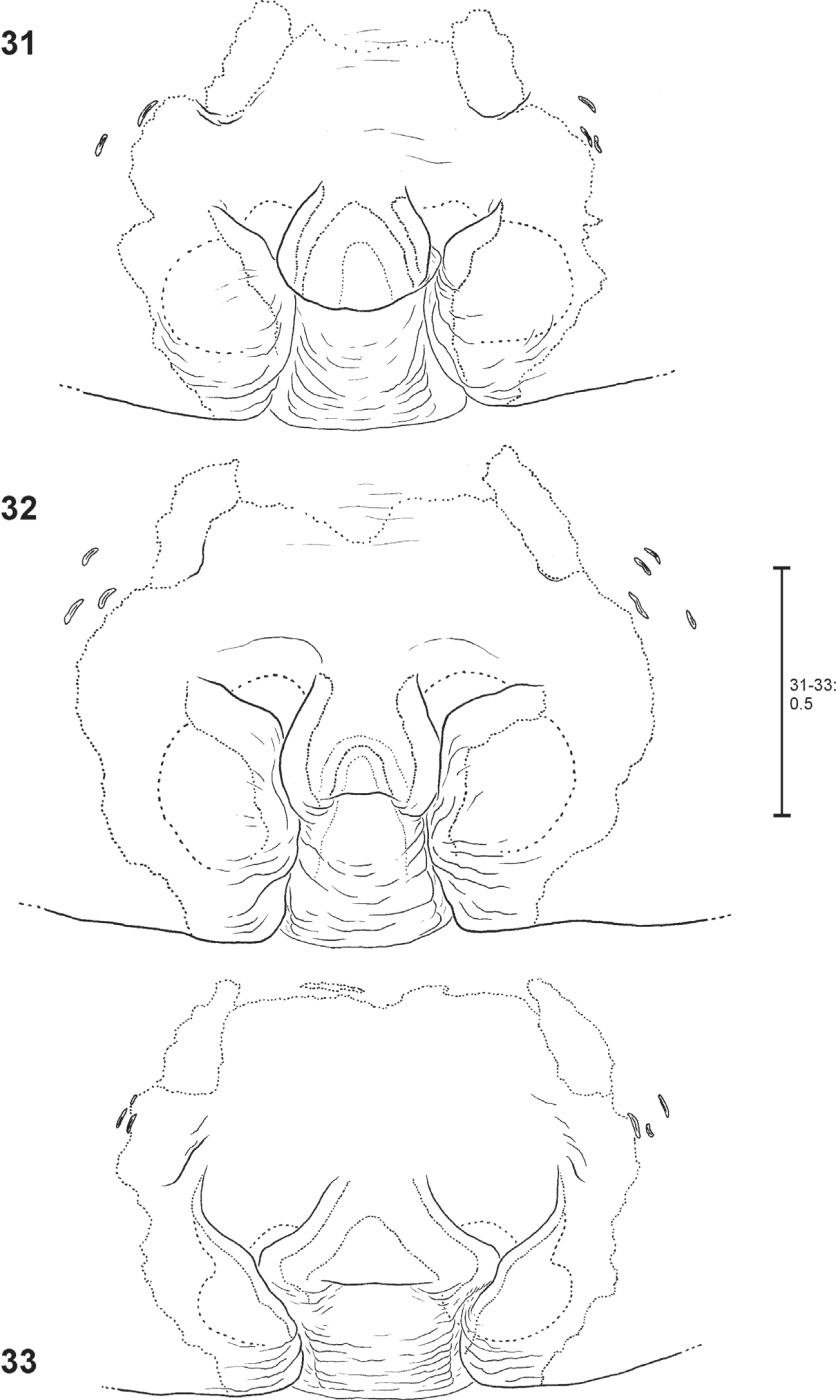
*Fecenia ochracea*, ♀ epigyne, ventral view **31** SB 430 from Southeastern Papua Prov., Indonesia **32** SB 98 from Mindiptana, Southeastern Papua Prov., Indonesia **33** SB 127 from Northern Sumatra, Indonesia.

**Figures 34–41. F9:**
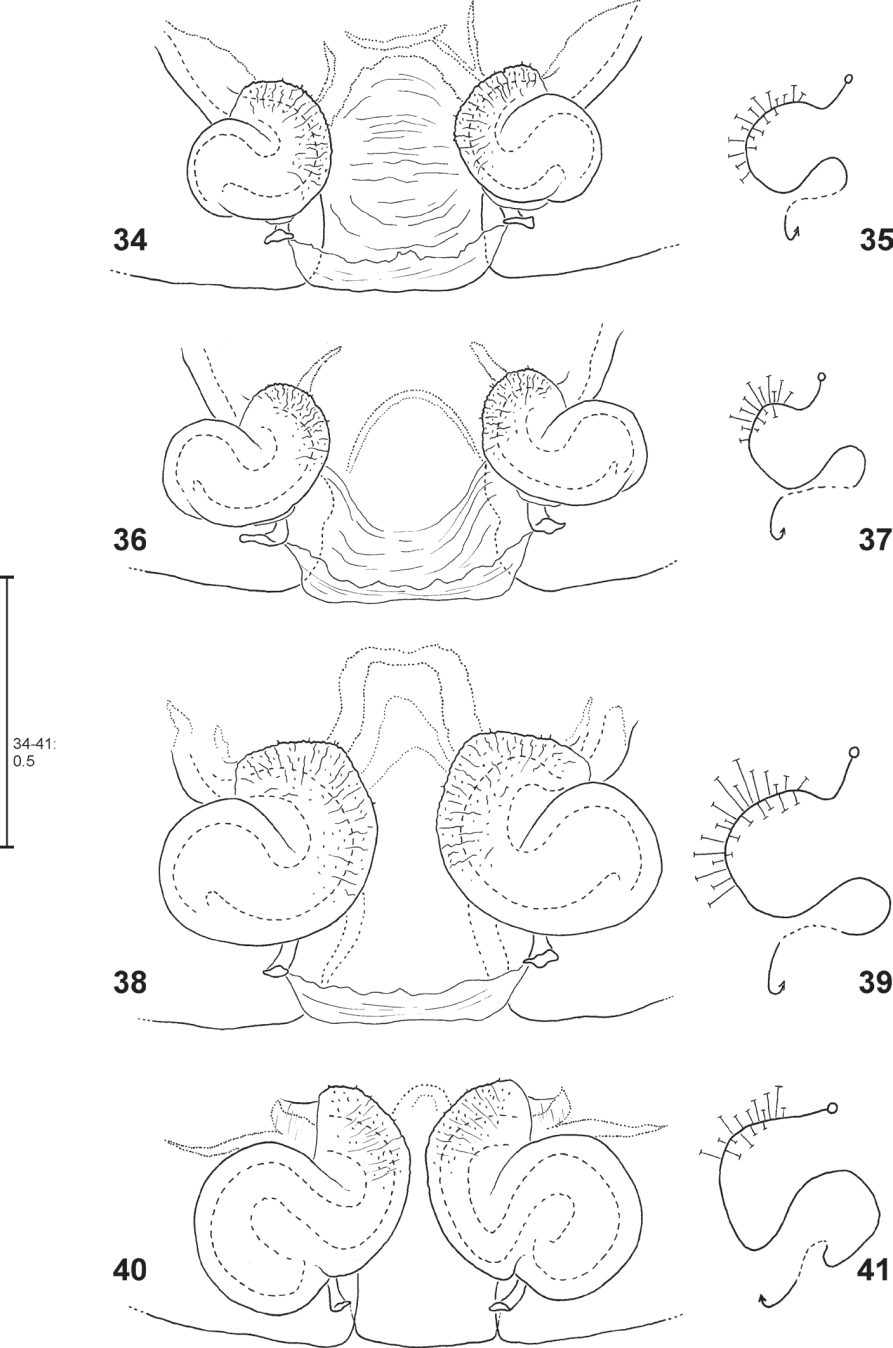
*Fecenia ochracea*, ♀ vulva, dorsal view **(34, 36, 38, 40)** with course of internal duct system **(35, 37, 39, 41). 34–35** Holotype of *Fecenia angustata* (SB 460) from Ternate, Indonesia. **36–37** Lectotype of *Fecenia buruana* (SB 418) from Buru, Indonesia. **38–39** Holotype of *Fecenia maforensis* (SB 464) from Northwestern Irian Jaya, Indonesia. **40–41** Holotype of *Fecenia cinerea* (SB 404) from Southern Papua Prov., Indonesia.

FEMALE(Measurements of holotype (SB 94) first, those of other specimens given as ranges in parentheses; Holotype misses both legs I as well as all limbs of legs IV from tibia on): Body and eye measurements. Carapace length 6.4 (3.2–6.9), carapace width 4.2 (2.2–4.3), anterior width of carapace 3.0 (1.7–3.1), opisthosoma length 9.1 (4.5–9.3), opisthosoma width 4.8 (2.2–5.2). Eyes: AME 0.33 (0.20–0.33), ALE 0.23 (0.15–0.23), PME 0.23 (0.15–0.23), PLE 0.25 (0.15–0.25), AME–AME 0.29 (0.22–0.29), AME–ALE 0.17 (0.09–0.17), PME–PME 0.36 (0.24–0.36), PME–PLE 0.48 (0.33–0.48), AME–PME 0.30 (0.15–0.30), ALE–PLE 0.24 (0.14–0.24), clypeus height at AME 0.44 (0.27–0.44), at ALE 0.42 (0.20–0.42). Measurements of palp and legs. Palp 6.3 (3.5–6.7) [2.2 (1.3–2.4), 1.1 (0.5–1.1), 1.2 (0.7–1.3), 1.8 (1.0–2.0)], I 17.2–40.7 [4.6–10.8, 1.3–2.9, 4.7.7–11.3, 4.5–11.4, 2.1–4.3], II 23.5 (10.8–24.5) [6.4 (3.0–6.6), 2.2 (1.1–2.2), 6.4 (2.9–6.8), 5.9 (2.50–6.2), 2.6 (1.3–2.7)], III 13.6 (6.6–14.6) [4.0 (1.9–4.3), 1.6 (0.8–1.7), 3.3 (1.5–3.5), 3.2 (1.5–3.4), 1.5 (0.9–1.7)], IV 10.0–21.4 [5.7 (2.7–5.8), 2.0 (1.0–2.1), 2.6–5.8, 2.5–5.3, 1.2–2.4]. Leg formula: 1243. Palpal claw with 10 (8–11) teeth. Spination (holotype from Ambon [except for leg I as well as tibia and metatarsus of leg IV, which are lost in holotype: spination of SB 474 from Ambon is listed instead]). Palp: 110, 000, 0000, 0000; legs: femur I 533(423), II 313, III 213, IV 111; patella I–IV 000; tibia I 3008, II 3006, III 0025, IV 2024; metatarsus I 2025, II 2025, III 1025, IV 1026. Copulatory organ: Anterior part of median septum (AS) of epigyne broad-“nose-like”, slightly broader than its posterior part (PS). Lateral lobes massive (Fig. 1, 102). Epigynal muscle sigillae (EM) mostly integrated in epigynal field (EF). Slit sense organs (SO) mostly outside EF. Vulva with relatively short and narrow transparent section of internal duct system (TSI). Strongly sclerotised section (SSI) compact, duct with two curves (Figs 2–3), apex of first one directed posterio-medially, of second anterio-laterally. Primordial copulatory organs: Pre-epigyne: Already strongly resembling the adult epigyne (Figs 20–21, 104). All major structures present in adult epigyne are recognizable in the pre-epigyne, too (of course much smaller). Epigynal field not or only poorly developed, EM far outside epigynal field (Fig. 21). Pre-pre-epigyne (antepenultimate instar): AML far shorter than in pre-epigyne and transversal ridge/edge of median septum (TR) hardly recognisable (Fig. 22, fine dotted line). Pre-vulva: Pre-receptacula bulbous/spherical and relatively close to each other. Distance between centres of pre-receptacula less than 3 times of diameter of one pre-receptaculum (Figs 23–24). Colouration: Male and female: As described for *Fecenia* in general, but white to beige patch in front of spinnerets may be rather unclear (Fig. 118), smaller or even absent. In one (SB 98, from Mindiptana, Eastern Papua Province, Indonesia) out of 103 specimens the light patches ventrally on opisthosoma are absent. Variation of copulatory organs: Among male specimens examined, cymbium differing at most slightly in length (Figs 11–18). In some specimens MA may be more massive (Fig. 10) or T slightly broader (Figs 9–10) than in others. Shape of prolatero-ventral lobe variable (Figs 7–10, 96). One specimen differing slightly more from the paralectotype of *Fecenia buruana* (Figs 8, 12, 16) from Buru island (which is the closest male record to the type locality, Ambon) than the others. This is SB 95 from Mindiptana, Eastern Papua Province of Indonesia: MA directed to 2:30-o'clock position (Fig. 9), embolus (E) slightly longer than in the other males examined, RTA broadest distally (Fig. 9). Additionally, T protruding a bit more out of cymbium (Fig. 13) than in the other specimens. In females intraspecific variation is higher. The shape of AS (Figs 1, 19, 27–33, 102) as well as the course of AML are highly variable. Number of SO varying among specimens without geographical dependence. Vulvae of the specimens examined show less variation than epigynes. The initial part of SSI may be slightly more prominent (Fig. 38). Further on, the position of SSI seems slightly shifted in some specimens (Figs 25, 34). Pre-epigynes also differing in shape of AS and in course of AML (Figs 20–21). Based on almost 80 females examined, all the variation described so far is neither geographically fixed, nor are there distinct forms of variants which recur here and there. In some cases females from exactly the same recording site show clear differences. And on the other hand females which are recorded in different localities, partly hundreds of km away from each other, look strikingly similar. Anyway, the following ‘form of females' has to be discussed separately (see remark below).

##### Remarks.

The vulvae of the holotype of *Fecenia cinerea* (SB 404) (Fig. 40) and the specimens recorded from Mindiptana, Eastern Papua Province of Indonesia (SB 96–98, 442) (SB 98 illustrated in Fig. 44) differ from all other females examined. The duct of SSI is somewhat longer, especially the second curve (Figs 40, 44). Consequently, the course of the internal duct system of these specimens (Figs 41, 45) differs from the remaining *Fecenia ochracea* females (Figs 3, 35, 37, 39, 43, 47). However, the vulvae of the holotype of *Fecenia cinerea* (SB 404) and female SB 98 do not correspond completely. In SB 404 the second curve of SSI protrudes more strongly in a lateral direction. In one specimen (SB 97, not illustrated) from Mindiptana the second curve of SSI is a bit shorter than in the others from this locality. The epigynes of SB 96-98, 404 and 442 differ in shape (SB 404: Fig. 28; SB 98: Fig. 32; others not illustrated). According to the differences in the shape of the vulvae (see above) it may be justified to revalidate *Fecenia cinerea* Hogg, 1914. However, the difference is little (second curve of SSI slightly longer than in *Fecenia ochracea*) and thus does not provide evidence for a clear species delimitation; especially considering that in one specimen from Mindiptana the second curve is again slightly shorter. In addition, if the females from Mindiptana should be regarded as *Fecenia cinerea*, then the male (SB 95, Figs 9, 13, 17), which was recorded from exactly the same locality, should be placed here, too. However, as discussed above, the palp structures of this male only slightly differ from the ones of other *Fecenia ochracea* males (though these differences are worth mentioning as intraspecific variation). Moreover, no males have been recorded from the type locality of *Fecenia cinerea* so far. Consequently, I refrain from changing the taxonomic status of *Fecenia cinerea*. More material from the type locality of *Fecenia cinerea*, especially males may enlighten this “problematic case”.

##### Disribution.

Philippines, Malaysia [Borneo], Indonesia [Sumatra, Borneo, Moluccas, West Papua, Java], Papua New Guinea, Solomon Islands, Australia [Northern Queensland].

#### Fecenia macilenta

(Simon, 1885)

http://species-id.net/wiki/Fecenia_macilenta

Figs 48–54 95, 101 114–115

Mezentia macilenta Simon, 1885: 451, pl. 10, fig. 17 (Description and illustration of ♂), [Holotype ♂ (SB 395) from MALAYSIA: Perak Prov. (Malacca): Region of Ipoh, Kinta river valley; M.J. de Morgan leg. 1884; MNHN AR5164, examined].
Fecenia macilenta - Simon 1887: 194 (Formal transfer from *Mezentia*, preoccupied by Stål, 1878 in Orthoptera, replacement name *Fecenia*); Simon 1892: 223, figs 171–172 (Illustration of ♂); Kulczyński 1908: 570; Reimoser 1936: 406; Lehtinen 1967: 234; Levi 1982: 136, figs 83–87, ad part, figs 84–85 (Illustration of ♂), figs 83, 86–87 misidentified; Murphy 1986: 65, figs 1–2 (Description and illustration of ♀); Coddington 1990: 10, fig. 18 (Illustration of ♂); Murphy and Murphy 2000: plate 21, fig. 6 (photo of ♀).

##### Additional material examined.

(1 ♂, 2 ♀♀). **MALAYSIA:** **Selangor Prov.:** Banting; W. Corley leg. VIII. 1981; 1 ♂ (SB389), 1 ♀ (SB 390), NHM. **INDONESIA:** **Sumatra: Sumatera Barat Prov.:** Panti (Road to Lubuk Sikaping & Bukittinggi), Rimba Panti Nature Reserve, primary rainforest; C. Deeleman leg.; 1 ♀ (SB 124), Deeleman Coll. in RMNH.

##### Diagnosis.

Males differ from other species by prominent dorso-retrolateral tibial apophysis (DRTA) (Figs 53–54, 95, 101) and slender and rather inconspicuous median apophysis (MA) (Fig. 53). Furthermore, ventral patellar apophysis (VPA) larger and retrolateral patellar apophysis (RPA) extending more clearly than in all other *Fecenia* species (Fig. 53–54, 95). Females distinguished from other species by epigyne with anterior margins of lateral lobes (AML) hardly visible and by posterior half of anterior part of median septum (AS) being distinctly darker than surrounding parts of epigyne (Fig. 114). Moreover, AS with permanent semicircular posterior half (Figs 48, 114).

##### Description.

MALE(Holotype (SB 395) is the largest of the males examined; consequently its measurements appear as maximum in each range. Eye measurements differ only insignificantly, so only those of the Holotype are listed): Body and eye measurements. Carapace length 5.4–5.8, carapace width 3.5–4.1, anterior width of carapace 2.3–2.7, opisthosoma length 5.4–7.4, opisthosoma width 2.8–3.2. Eyes: AME 0.47, ALE 0.34, PME 0.31, PLE 0.29, AME–AME 0.27, AME–ALE 0.08, PME–PME 0.35, PME–PLE 0.40, AME–PME 0.15, ALE–PLE 0.19, clypeus height at AME 0.67, at ALE 0.54. Measurements of palp and legs. Palp 7.3–8.5 [2.8–3.3, 1.2–1.4, 1.1–1.3, 2.2–2.5], I 53.5–67.4 [14.7–18.8, 2.3, 15.3–19.2, 16.7–19.9, 4.5–7.2], II 27.9–35.0 [7.5–9.4, 1.9–2.2, 7.7–9.7, 7.7–9.9, 3.1–3.8], III 15.9–19.4 [4.6–5.6, 1.5–1.7, 3.8–4.8, 4.1–4.9, 1.9–2.4], IV 25.1–30.9 [6.8–8.3, 1.7–2.0, 6.4–8.2, 7.4–8.9, 2.8–3.5]. Leg formula: 1243. Male chelicerae differing from general appearance of *Fecenia*: Basal limb ca. 4 times longer than broad. Spination (holotype from Kinta river, Malaysia). Palp: without any spines; legs: femur I 410(300), II 100, III 010, IV 001; patella I–IV 000; tibia I–II 2004, III 0000(0001), IV 0013; metatarsus I 3014(1014), II 1015, III 1015(1014), IV 1015. Copulatory organ: Ventral patellar apophysis (VPA) arising centrally on palpal patella (Figs 52, 54), RTA small, less than 1/3 of the length of the massive DRTA. Median apophysis (MA) distally slightly bent. General direction of MA is 12:30 or 1:00-o'clock. Embolus (E) arising in ca. 6:30-o'clock-position on tegulum (T), broader than in all other *Fecenia* species and almost twice as long as width of T. The latter slightly longer than broad. Membranous process (MP) of tegulum directed proximally (Figs 53, 95). Conductor (C) longer than MA, arising centrally in upper third of T.

FEMALE (The two females examined differ not or only marginally in almost all measurements, so only those of SB 390 are listed, except for opisthosoma measurements [ranges, SB 390 from Banting, Malaysia first]): Body and eye measurements. Carapace length 5.3, carapace width 3.7, anterior width of carapace 3.0, opisthosoma length 7.6–8.3, opisthosoma width 4.5–4.9. Eyes: AME 0.34, ALE 0.27, PME 0.27, PLE 0.22, AME–AME 0.26, AME–ALE 0.12, PME–PME 0.34, PME–PLE 0.46, AME–PME 0.22, ALE–PLE 0.24, clypeus height at AME 0.44, at ALE 0.41. Measurements of palp and legs. Palp 5.9 [2.0, 1.0, 1.0, 1.9], I 30.2 [8.1, 2.3, 8.5, 7.9, 3.4], II 19.7 [5.4, 1.9, 5.3, 4.7, 2.4], III 12.4 [3.6, 1.5, 2.9, 2.8, 1.6], IV 17.7 [4.9, 1.7, 4.6, 4.4, 2.1]. Leg formula: 1243. Palpal claw with 12 teeth (Fig. 51). Copulatory organ: Anterior part of median septum (AS) of epigyne “nose-like”, broader than posterior part (PS) (Fig. 48). Lateral lobes voluminous (Figs 48, 114). Epigynal muscle sigilla (EM) integrated in epigynal field. Slit sense organs (SO) outside epigynal field. Vulva with voluminous sclerotised section of internal duct system (SSI) (Fig. 49) and short and narrow transparent section (TSI), which is in dorsal view partly covered by SSI. The latter almost in contact with each other. Duct of SSI with three curves (Fig. 50). Fertilisation duct (FD) narrow. Colouration: Male and female: As described for *Fecenia* in general, but white to beige patch in front of spinnerets in one female rather unclear and smaller. Variation of copulatory organs: Males varying only insignificantly. Females: In female from Banting, Malaysia (SB 390) dark section of AS reaching further anteriorly (dotted line within AS in Fig. 48) than in SB 124 (Fig. 114). Number of SO among specimens varies from two to four. Distance between SSI slightly longer in SB 124 (Fig. 115).

##### Disribution.

Malaysia, Indonesia [Sumatra].

**Figures 42–47.**
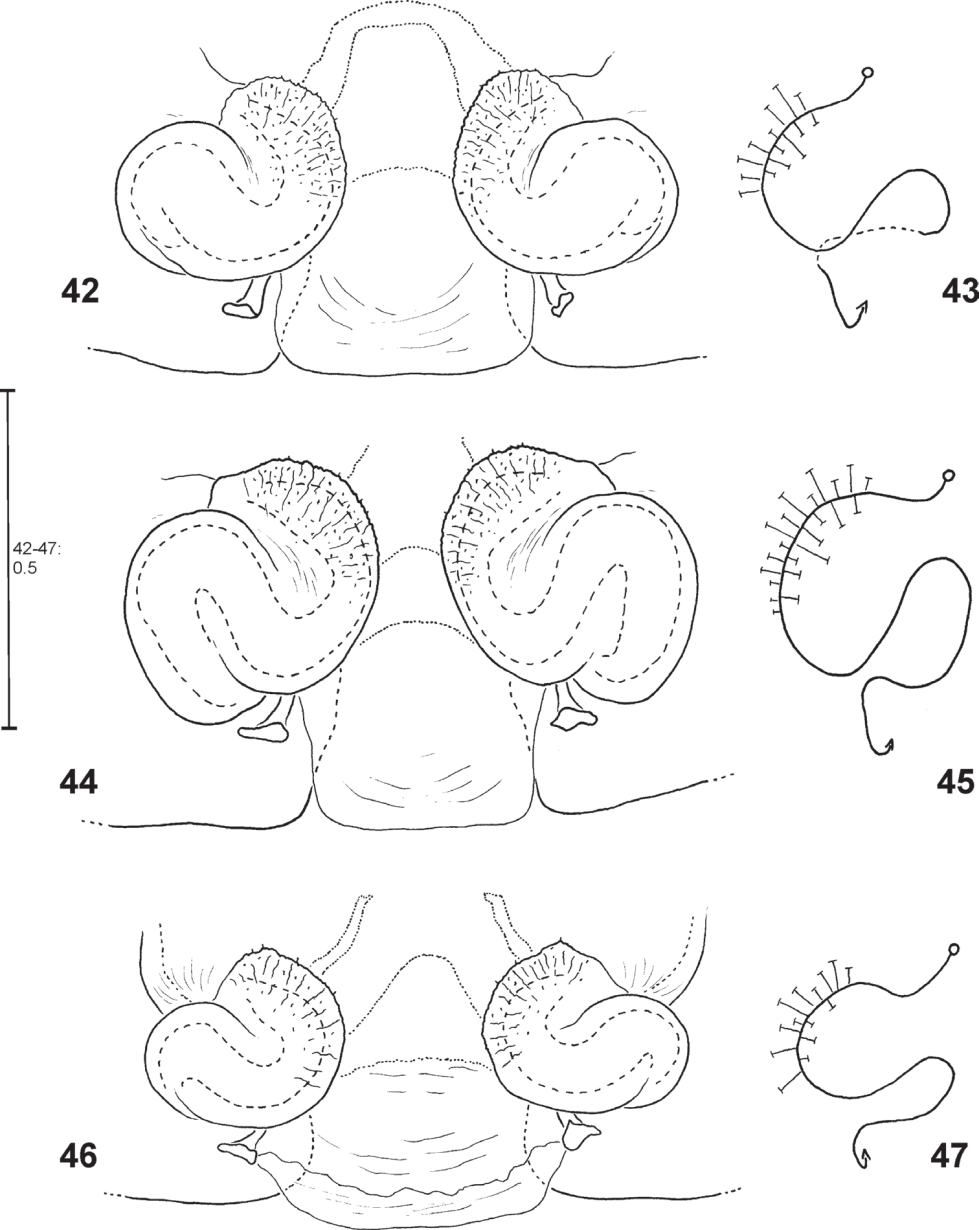
*Fecenia ochracea*, ♀ vulva, dorsal view **(42, 44, 46)** with course of internal duct system **(43, 45, 47). 42–43** SB 430 from Southeastern Papua Prov., Indonesia. **44–45** SB 98 from Mindiptana, Southeastern Papua Prov., Indonesia. **46–47** SB 127 from Northern Sumatra, Indonesia.

#### Fecenia protensa

Thorell, 1891, stat. n.

http://species-id.net/wiki/Fecenia_protensa

Figs 55 [Fig F13] –70 98–99 108–110 119

Fecenia protensa Thorell, 1891: 31 (Description of immature ♀), [Holotype s.a. ♀ (SB 620) from INDIA: Nicobar Islands: Nancowry; Bille, Kjellerup, Behn and Reinhardt leg. 1845–1847, Galathea Expedition; ZMUC 13091, examined]; Kulczyński 1908: 570; Reimoser 1936: 406; Lehtinen 1967: 234; Levi 1982: 136 (Synonymy with *Fecenia macilenta*, rejected); Murphy 1986: 65 (Statement concerning synonymy with *Fecenia macilenta*).
Fecenia sumatrana Kulczyński, 1908: 568, pl. 23, fig. 20 (Description of ♀), [Holotype ♀ (SB 357) from INDONESIA: Sumatra: Lampung Prov.: Palembang; Dr S. Libelt leg.; MIZ 212∙322, examined]; Reimoser 1929: 132 (Listing of first record from Mentawai islands); Reimoser 1936: 407; Lehtinen 1967: 234 (Synonymy with *Fecenia macilenta*, rejected by subsequent author); Murphy 1986: 65 (Synonymy with *Fecenia travancoria*, rejected). Syn. n.
Psechrus nicobarensis Tikader, 1977: 208, fig. 27A–E (Description and illustration of ♂ and ♀), [Holotype ♀ as well as 8 ♀♀ paratypes and 2 ♂♂ paratypes from INDIA: West Bengal Province: Nicobar Islands, Car-Nicobar Isl., Kakana village; B.K. Tikader leg. 9.III.1970; NZSI, not available on request, thus not examined]; Jose and Sebastian 2001: 304 (genus name misapplied). Syn. n.
Fecenia nicobarensis - Levi 1982: 138 (Transfer from *Psechrus*).
Fecenia macilenta - Levi 1982: 136, figs 83–87, ad part, figs 83, 86–87 misidentified (figs 83 and 87: Illustration of s.a. ♀ and ♀); Koh 1989: 76, fig. embedded in text, misidentified (Illustration of ♀).
Psechrus alticeps - Jose and Sebastian 2001: 304, fig. 1, misidentified. Note: Jose and Sebastian (2001) copied the illustrations of fig. 27 in Tikader (1977), pasted them in their fig. 1 and partly modified them. In their fig. 1a they changed the eye arrangement and colour pattern of the carapace as well as the colour pattern of the opisthosoma “*Psechrus*”-like. Their figs 1b and 1g show the female and male copulatory organs of *Fecenia protensa* after Tikader (1977, there sub *Psechrus nicobarensis*). Their fig. 1f is a misinterpretation and definitely shows neither the vulva of *Fecenia protensa* nor the one of *Psechrus alticeps* Pocock, 1899, which is a junior synonym of *Psechrus torvus* (O. Pickard-Cambridge, 1869) (Levi 1982).

##### Additional material.

(6 ♂♂, 38 ♀♀, 2 s.a. ♂♂, 5 s.a. ♀♀, 10 juvenile specimens). **THAILAND:** **Nakhon Nayok Prov.:** Khao Yai N.P. located ca. 120 km North-East of Bangkok, evergreen tropical rainforest, ca. 150 m; P. Hillyard leg. 12.III.1984; 1 ♀ (SB 393), NHM. Khao Yai N.P., forests surrounding Park Headquarters, 800-900 m; P. Dankittipakul leg. 15.XI.2006; 1 ♂ (SB 218), MHNG. **Nakhon Ratchasima Prov.:** Khao Yai N.P., rainforest; C.L. and P.R. Deeleman leg. 28.XII.1988; 1 ♂ (SB 128, died directly after adult moult), Deeleman Coll. in RMNH. **Chantaburi Prov.:** Chantaburi Distr.: Nam Tok Phliu N.P., 50 m; P. Schwendinger leg. 11.IX.1993; 1 ♂ (SB 136), MHNG. **Trat Prov.:** Koh Chang Isl.: Khlong Chao Luam, 12°06'30"N, 102°17'49"E, 30–150 m, secondary forest along stream, in shrubs; P. Jäger and S. Bayer leg. 3.XI.2009; 1 ♂ (SB 512), 1 ♀ (SB 458), 2 juvs (SB 328, 350), SMF. **Surat Thani Prov.:** Khao Nan N.P.; P. Dankittipakul leg. 17.VIII.2006; 1 ♀ (SB 202), MHNG; P. Dankittipakul leg. 27.X.2006; 1 ♀ (SB 206), SMF. **Krabi Prov.:** Krabi Distr.: Thab Khaek, Hang Nak Hill Nature Trail, 8°05'43"N, 98°45'11"E, 300 m, semi-evergreen rainforest; P. Schwendinger leg. 6.VI.2009; 1 ♀ (SB 195), SMF. **Phuket Prov.:** Phuket, Ton Sai Waterfall, 8°01'N, 98°25'E, 150–200 m; M. Andersen, O. Martin and N. Scharff leg. 12.X.1991; 3 ♀♀ (SB 888–890), 1 s.a. ♂ (SB 891), ZMUC 4536. **Song Khla Prov.:** Khao Khor Hong, a small mountainous area behind Prince of Song Khla University campus; B. Phongsee leg. 15.IX.2005; 1 ♀ (SB 215), 1 s.a. ♀ (SB 216), 3 juvs (SB 782–784), MHNG. **MALAYSIA:** **Pahang Prov.:** Cameron Highlands at Tanah Rata, 4°28'N, 101°23'E; V. and B. Roth leg. 14.-20.IV.1990; 1 ♀ (SB 184), 1 s.a. ♀ (SB 185), USNM, 1 ♀ (SB 949), CAS ENT9032226. **Selangor Prov.:** Gombak, field station, forest; C.L. Deeleman leg. 6.VII.1992; 1 ♀ (SB 117); C.L. Deeleman and J.C. van Kempen leg. 2.VII.1992, by night; 1 ♀ (SB 112), both Deeleman Coll. in RMNH. **Borneo: Sarawak Prov.:** Northern Sarawak; P. Nabawi leg.; [ex Coll. Wunderlich]; 1 ♀ (SB 1142), SMF. Gunong Mulu N.P., Environs Camp I, 1150 m, shrubs; F. Wanless leg. by net sweeping, R.G.S. Mulu Exped. 1977–78; 1 ♀ (SB 391), NHM. Gunong Mulu N.P., rain forest; C.L. and P.R. Deeleman leg. 4.X.2003; 1 ♀ (SB 131), s.a. ♀ (SB 897), 1 juv. (SB 898), Deeleman Coll. in RMNH. Kuching: Matang Reserve, primary forest, big old tree in clearing; C.L. and P.R. Deeleman leg. 25.III.1986; 2 ♀♀ (SB 107–108), 1 s.a. ♂ (SB 899), 3 juvs (SB 900–902), Deeleman Coll. in RMNH. **Sabah Prov.:** Kinabalu N.P., Poring Hot Springs, 5°02'N, 116°42'E, 600–700 m, primary forest; A. Floren leg. 3.III.1996 by canopy fogging “Ridge”; 1 ♀ (SB 519), Deeleman Coll. in RMNH. **SINGAPORE:** Singapore (no further details); H.N. Ridley leg.; 7 ♀♀ (SB 408–414), 1 s.a. ♀ (SB 407), NHM. Singapore: Sime Road: secondary forest; J. Koh leg. 1998; 1 ♀ (SB 186), USNM. **INDONESIA:** **Sumatra: Nanggroe Aceh Darussalam Prov.:** Simeulue Isl.; E. Jacobson leg.; 1 s.a. ♀ (SB 462), NHMW 12387. **Sumatera Barat Prov.:** Lubuk Sikaping, Panti Reserve; C.L. and P.R. Deeleman leg. 14.VIII.1982; 1 ♀ (SB 125), Deeleman Coll. in RMNH. **Borneo: Kalimantan Timur Prov.:** 40 km NNW of Balikpapan, Sepaku, isolated stand of rainforest; C.L. and P.R. Deeleman leg. 5.VIII.1980; 1 ♀ (SB 126), Deeleman Coll. in RMNH. **Bali Prov.:** Air Terjung Waterfall, c/o Munduk, 8°15'27.8"S, 115°04'14.1"E, ca. 1000 m; S. Huber leg. 11.IV.2011; 5 ♀♀ (SB 1013–1017), SMF. Ubud, 8°29'51"S, 115°15'18.4"E, ca. 330 m; S. Huber leg. 30.III.2011; 1 ♀ (SB 1028), SMF. Candi Dasa, creek forest, 8°30'13"S, 115°33'47"E; S. Huber leg. 16.–20.III.2009; 1 ♂ (SB 137), 2 ♀♀ (SB 196, 256), 1 juv. (SB 906), SMF. **Nusa Tenggara Barat Prov.:** Flores Isl., East of Labuan Bajo, rainforest, 8°33'60"S, 120°00'02"E; S. Huber leg. 24.III.2009; 1 ♂ (SB 196, only left palp and a few body parts remained), SMF.

##### Diagnosis.

Females distinguished from other *Fecenia* species except *Fecenia travancoria* by having anterior margins of lateral lobes (AML) anteriorly more or less converging and surrounding epigynal pit partly and the anterior part of median septum (AS) comprising a longitudinal, anteriorly pointed folding (Fig. 55); moreover, by having a notched transversal edge (TR) of median septum. Distinguished from *Fecenia travancoria* by borderline (BL) between strongly sclerotised section of internal duct system (SSI) and transparent section (TSI) running almost transversal (Fig. 56). In males RTA short, at most ½ the width of palpal tibia, and knobbed, almost as broad as long (Figs 62–63, 98). Median apophysis (MA) almost semicircular and shorter than width of tegulum (T) (Fig. 62, 98). In contrast to the similar male of *Fecenia cylindrata* embolus (E) without basal apophysis (bEA) and ventral patellar apophysis (VPA) arising proximally on patella (Figs 60–61, 99).

##### Description.

MALE:Body and eye measurements. Carapace length 3.1–4.4, carapace width 2.1–3.0, anterior width of carapace 1.4–1.9, opisthosoma length 4.1–6.4, opisthosoma width 1.6–2.6. Eyes: AME 0.25–0.27, ALE 0.17–0.18, PME 0.18–0.19, PLE 0.17–0.19, AME–AME 0.14–0.22, AME–ALE 0.07–0.19, PME–PME 0.18–0.25, PME–PLE 0.25–0.34, AME–PME 0.13–0.19, ALE–PLE 0.10–0.18, clypeus height at AME 0.29–0.35, at ALE 0.21–0.26. Measurements of palp and legs. Palp 4.0–5.2 [1.5–2.1, 0.7–0.9, 0.5–0.7, 1.3–1.6], I 34.7–52.5 [9.1–14.2, 1.4–2.1, 9.5–14.6, 10.6–16.2, 4.1–5.4], II 16.3–25.1 [4.3–6.7, 1.2–1.6, 4.5–7.0, 4.3–7.0, 2.0–2.8], III 9.2–14.0 [2.6–4.0, 0.9–1.3, 2.2–3.6, 2.3–3.4, 1.2–1.7], IV 15.5–23.1 [4.1–6.3, 1.1–1.6, 4.1–6.2, 4.4–6.5, 1.8–2.5]. Leg formula: 1243. Copulatory organ. Retrolateral patellar apophysis (RPA) rather inconspicuous (Figs 61–63, 98–99). Median apophysis (MA) with tip in ca. 1:00-o'clock-position (Fig. 62) and in retrolateral view almost straight (Figs 61, 99). Embolus (E) arising in ca. 7:30-o'clock-position on tegulum (T), distally thicker than in *Fecenia cylindrata* and at most as long as width of T. The latter almost round. Membranous process of tegulum (MP) reaches far up, mostly 10:00–10:30-o'clock-position. Conductor (C) small, size and shape similar like in *Fecenia ochracea*, arises medially in upper third of T.

FEMALE: Body and eye measurements. Carapace length 3.3–4.7, carapace width 1.9–3.1, anterior width of carapace 1.6–2.3, opisthosoma length 5.5–8.1, opisthosoma width 2.4–4.2. Eyes: AME 0.22–0.28, ALE 0.15–0.21, PME 0.17–0.22, PLE 0.16–0.19, AME–AME 0.19–0.26, AME–ALE 0.10–0.14, PME–PME 0.23–0.29, PME–PLE 0.29–0.40, AME–PME 0.23–0.24, ALE–PLE 0.16–0.19, clypeus height at AME 0.33–0.41, at ALE 0.29–0.38. Measurements of palp and legs. Palp 3.5–5.1 [1.2–1.7, 0.6–0.9, 0.6–1.0, 1.1–1.5], I 19.2–26.8 [4.8–7.0, 1.5–2.0, 5.4–7.8, 5.1–7.2, 2.4–2.8], II 11.3–16.5 [3.0–4.3, 1.1–1.6, 3.1–4.7, 2.7–4.0, 1.4–1.9], III 7.1–10.6 [2.1–3.1, 0.8–1.3, 1.8–2.6, 1.5–2.3, 0.9–1.3], IV 10.7–16.0 [2.8–4.4, 1.1–1.7, 3.1–4.6, 2.5–3.6, 1.2–1.7]. Leg formula: 1243. Palpal claw with 8–11 teeth. Spination (immature holotype of *Fecenia protensa* from Nicobar Islands in poor condition! spination of holotype of *Fecenia sumatrana* from Palembang, Sumatra listed instead). Palp: 110, 110, 0100, 1004 (spines on patella, tibia and tarsus with only half the size as those of femur!); legs: femur I 310, II 320, III 011, IV 020; patella I–IV 000; tibia I–II 3006, III 0023, IV 0024; metatarsus I 2025, II–III 2015, IV 1015. Copulatory organ: Epigyne in general appearance characteristically rounded-“W”-shaped (Figs 55, 64, 108). AML mostly strongly sclerotised, converging anteriorly and surrounding epigynal pit partly. AS clearly broader than PS. Epigynal muscle sigilla (EM) integrated in epigynal field or at least located very close by, same for slit sense organs (SO) (Figs 55, 64). Vulva with medium sized (longer than in *Fecenia ochracea*, *Fecenia macilenta* and *Fecenia travancoria*, shorter than in *Fecenia cylindrata*) and broad TSI (56, 65, 109). SSI more slender than in all other *Fecenia* species, duct with 2–3 curves (Fig. 57). Primordial copulatory organ: Pre-epigyne: “Crown”-like (Figs 58, 66, 69, 110). Primordium of AS already recognisable, broad “W”-like. Epigynal field not or only poorly developed, EM far outside epigynal field (Fig. 69). Pre-pre-epigyne: Prongs of the “crown” small (Fig. 70). Pre-vulva: Pre-receptacula bulbous/spherical (Figs 59, 67–68). Distance between centres of pre-receptacula more than three times diameter of one pre-receptaculum. Variation of copulatory organs: Cymbium length of male palp differing slightly among specimens examined (Figs 60–63, 98–99), MA may be extending further beyond retrolateral cymbium margin (Figs 63, 98). T in some specimens slightly broader (Fig. 98) than in others. RPA may be slightly larger (Fig. 63) than in general. In females the shape of AS may vary, e.g. the posterior notch is larger and the anteriorly pointed longitudinal folding is as such hardly recognisable (Fig. 64). Number of SO among specimens varying without geographical dependence. TSI varying in length (Fig. 56, 65). In dorsal view BL direction of vulva varies from 8:30 (Fig. 65) to almost 9:30-o'clock-position (Fig. 56). Pre-epigynes differing in shape of TR (Figs 58, 66). The most frequent shape seems to be the one of SB 216 (Fig. 69) and holotype SB 620 (Figs 58, 110). Number of SO varying strongly.

Pre-vulva may be slightly more structured (Fig. 67).

##### Remarks.

The reasons for revalidation of *Fecenia protensa* and the synonymy of *Fecenia sumatrana* with the former are as follows: In Thailand, Malaysia, Singapore and on Bali at several localities subadult *Fecenia* females were collected together with adult females respectively, which showed the characteristic rounded-“W”-shaped epigyne. The pre-epigyne of the subadult female holotype of *Fecenia protensa* (SB 620) matches the ones of the subadult females mentioned above. In 1908 Kulczyński described *Fecenia sumatrana.* The (adult) female holotype of this species exhibits the characteristic rounded-“W”-shaped epigyne. The adult females mentioned above match the holotype of *Fecenia sumatrana*. *Fecenia protensa* is the oldest name available and hence the valid name for this taxonomical species. It is distinguished from *Fecenia travancoria* by the BL of the vulva running almost transversal. Consequently, *Fecenia sumatrana* is not a junior synonym of *Fecenia travancoria* as postulated in Murphy (1986), but a junior synonym of *Fecenia protensa*. Both, *Fecenia protensa* and *Fecenia travancoria* are regarded as valid species (see also remarks sub species description of *Fecenia travancoria*).

Reason for synonymy of *Fecenia nicobarensis* with *Fecenia protensa*: Although the types of *Psechrus nicobarensis* were not available on request it became obvious that Tikader (1977) dealt with *Fecenia protensa*. The drawing of the female epigyne in Tikader (1977: 208, fig. 27B) is not very informative, however, the rounded-“W”-shaped character of the epigyne is very clear. His fig. 27E of the right male palp is more detailed. However, the proportions probably do not reflect the real situation. Additionally, this illustration does not represent an exact ventral view of the palp. If the left palps of the males examined herein (SB 128, 136, 137, 218, 219, 512) were arranged in the same way/position, they would match the (mirrored) drawing in Tikader (1977).

##### Disribution.

India [Nicobar Islands], Thailand, Malaysia, Singapore, Indonesia [Sumatra, Borneo, Bali].

**Figures 48–54.**
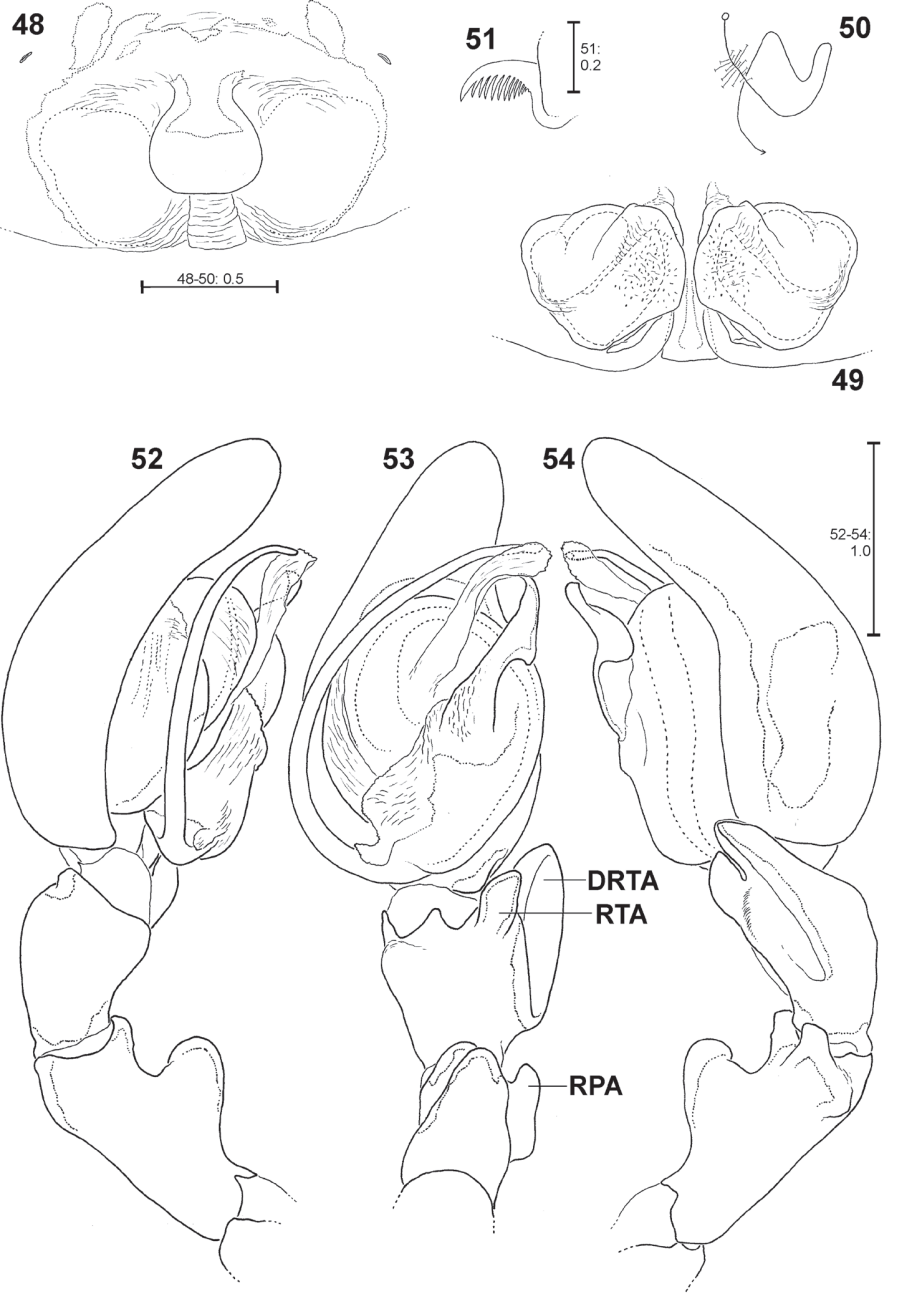
*Fecenia macilenta*. **48–51** ♀ (SB 390) from Selangor Prov., Malaysia. **52–54** Holotype ♂ (SB 395) from Perak Prov., Malaysia. **48** Epigyne, ventral view. **49** Vulva, dorsal view. **50** Course of internal duct system. **51** Left palpal claw, retrolateral view. **52–54** left palp, prolateral **(52)**, ventral **(53)** and retrolateral **(54)** view. DRTA = Dorso-retrolateral tibial apophysis; RPA = Retrolateral patellar apophysis; RTA = Retrolateral tibial apophysis.

**Figures 55–59.**
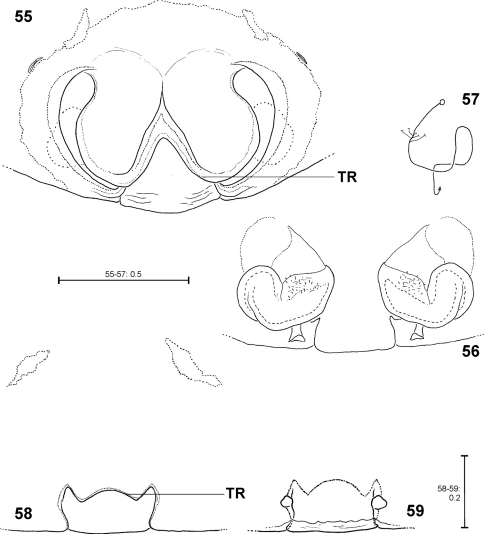
*Fecenia protensa*, ♀ copulatory organ/primordial copulatory organ **55–57** Holotype ♀ of *Fecenia sumatrana* (SB 357) from Southern Sumatra, Indonesia **58–59** Holotype s.a. ♀ (SB 620) of *Fecenia protensa* from Nicobar Islands, India **55** Epigyne, ventral view **56** Vulva, dorsal view **57** Course of internal duct system **58** Pre-epigyne, ventral view **59** Pre-vulva, dorsal view. TR = Transversal edge/ridge of median septum (**55** in epigyne, **58** corresponding structure in pre-epigyne).

**Figures 60–63. F13:**
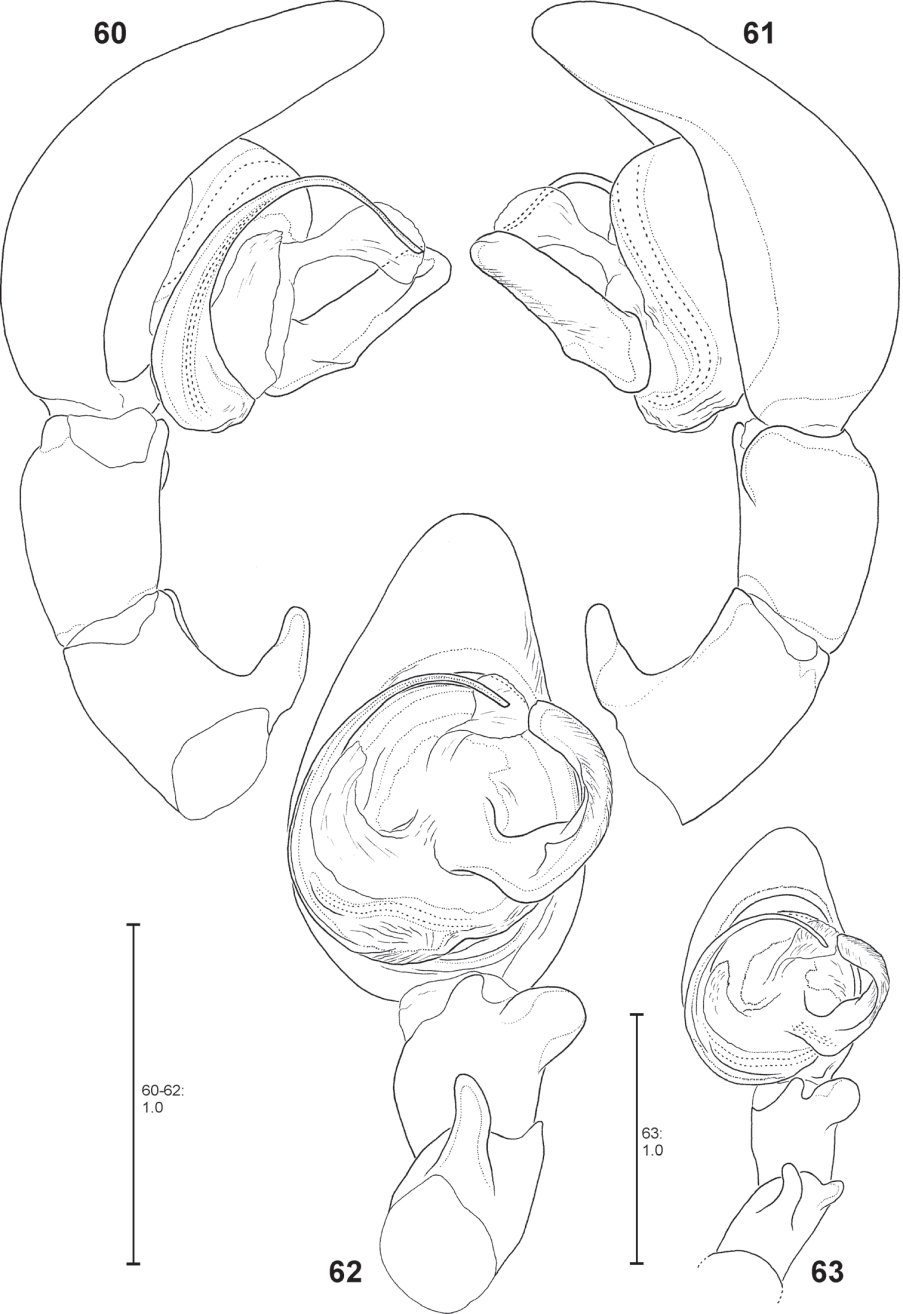
*Fecenia protensa*, ♂ palp **60–62** SB 218 from Nakhon Ratchasima Prov., Thailand **63** SB 137 from Bali, Indonesia **60** Prolateral view. **61** Retrolateral view **62–63** Ventral view.

**Figures 64–70.**
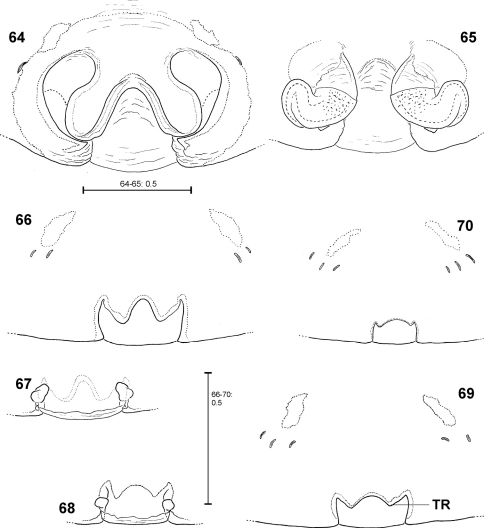
*Fecenia protensa*, ♀ copulatory organ/primordial copulatory organ **64–65** ♀ SB 410 from Singapore **66–67** s.a. ♀ SB 185 from Pahang Prov., Malaysia **68–69** s.a. ♀ SB 216 from Songkhla Prov., Thailand **70** p.s.a. ♀ SB 897 from Sarawak Prov., Malaysia **64** Epigyne, ventral view **65** Vulva, dorsal view **66, 69** Pre-epigyne, ventral view **67–68** Pre-vulva, dorsal view **70** Pre-pre-epigyne, ventral view. TR = Transverse edge/ridge of (in this case primordial) median septum.

**Figures 71–78.**
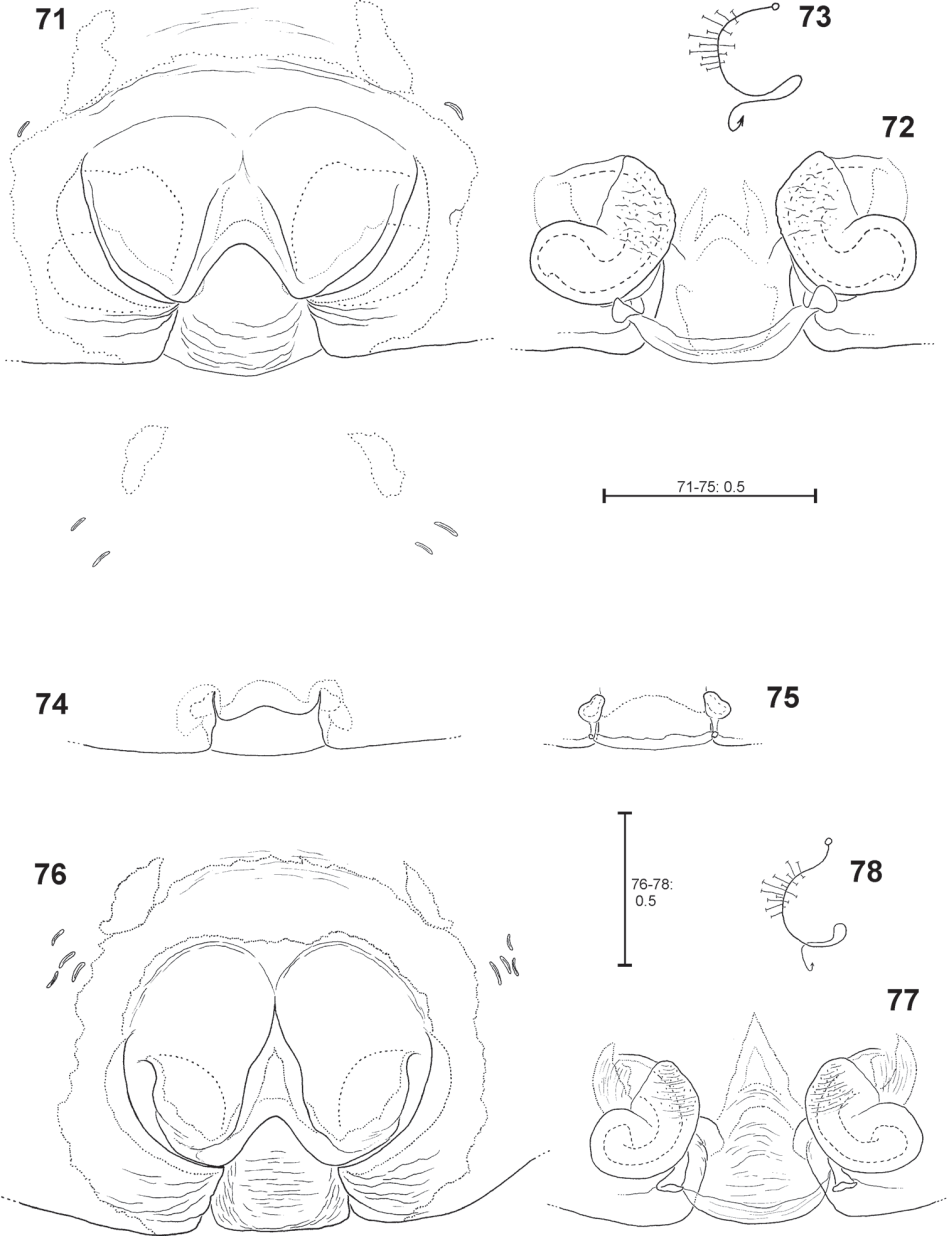
*Fecenia travancoria*, ♀ copulatory organ/primordial copulatory organ **71–73** ♀ SB 118 **74–75** s.a. ♀ SB 119, both from Erawan, Kanchanaburi Prov., Thailand **76–78** Holotype ♀ (SB 403) from Kerala Prov., India **71, 76** Epigyne, ventral view **72, 77** Vulva, dorsal view **73, 78** Course of internal duct system **74** Pre-epigyne, ventral view. **75** Pre-vulva, dorsal view.

#### Fecenia travancoria

Pocock, 1899

http://species-id.net/wiki/Fecenia_travancoria

Figs 71–78 111–113

Fecenia travancoria Pocock, 1899: 750 (Description of ♀), [Holotype ♀ (SB 403) from INDIA: Kerala Prov.: Madatory; H. Ferguson leg. III.1896; NHM 99·1·17·36, examined]; Kulczyński 1908: 570; Reimoser 1936: 406; Lehtinen 1967: 234 (Synonymy with *Fecenia macilenta*); Murphy 1986: 65 (Removed from synonymy with *Fecenia macilenta*); Jose and Sebastian 2001: 304; Sebastian and Peter 2009: 277 (Description of ♀).
Fecenia macilenta - Levi 1982: 136, figs 83–87, ad part, figs 83, 86–87 misidentified (fig 86: Illustration of ♀).

##### Additional material examined.

(3 ♀♀, 2 s.a. ♀♀, 2 juvenile specimens). **INDIA:** **Kerala Prov.:** Ernakulam; K. S. Jose leg. 23.III.2001; 1 ♀ (SB 863, checked via photo of entire specimen, ventral view, kindly provided by K. S. Jose), SJPC. **SRI LANKA:** **Sabaragamuwa Prov.:** Ratnapura, peak wilderness area; W. Sedgwick leg. 11.VIII.1979; 1 juv. (SB 481), MCZ 82528. Pitadeniya, Sinharaja Nature Reserve, 6°21'40.2"N, 80°29'03.6"E, ca. 300 m, primary forest, in palm, 1.5 m above ground; V. Hartmann leg. 16.I.2011 as immature, raised in laboratory, adult 05.IV.2011; 1 ♀ (SB 982, from this specimen the exuviae of the subadult instar, thus its pre-epigyne, was kept and preserved), SMF. **THAILAND:** **Kanchanaburi Prov.:** Erawan Waterfall in Erawan N.P., evergreen rainforest; C.L. & P.R. Deeleman leg. 15.III.1986; 1 ♀ (SB 118), 1 s.a. ♀ (SB 119), 2 juvs (SB 903–904), Deeleman Coll. in RMNH.

##### Diagnosis.

Females distinguished from other *Fecenia* species except *Fecenia protensa* by having anterior margins of lateral lobes (AML) anteriorly more or less converging and surrounding epigynal pit partly and the anterior part of median septum (AS) comprising a longitudinal, anteriorly pointed folding (Fig. 76); moreover, by having a notched transversal edge (TR) of median septum. Females are distinguished from *Fecenia protensa* by the almost longitudinal borderline (BL) between strongly sclerotised section (SSI) and the transparent section of internal duct system (TSI) in vulva (Fig. 77).

##### Description.

MALE: unknown.

FEMALE(measurements of holotype first, those of other females in parentheses): Body and eye measurements. Carapace length 5.9 (4.4–5.2) , carapace width 4.0 (3.0–3.3), anterior width of carapace 2.9 (2.2–2.5), opisthosoma length 7.8 (7.2–12.3), opisthosoma width 4.3 (4.0–5.4). Eyes: AME 0.36 (0.23–0.28), ALE 0.20 (0.12–0.18), PME 0.24 (0.14–0.21), PLE 0.23 (0.14–0.20), AME–AME 0.37 (0.22–0.31), AME–ALE 0.15 (0.09–0.13), PME–PME 0.47 (0.26–0.39), PME–PLE 0.48 (0.39–0.43), AME–PME 0.37 (0.22–0.30), ALE–PLE 0.26 (0.21–0.24), clypeus height at AME 0.43 (0.36–0.40), at ALE 0.39 (0.32–0.34). Measurements of palp and legs. Palp 6.2 (4.5–5.7) [2.1 (1.5–1.9), 1.1 (0.8–1.0), 1.1 (0.8–1.0), 1.9 (1.4–1.8)], I 33.3 (24.4–29.7) [8.8 (6.4–7.9), 2.4 (1.9–2.1), 9.0 (7.1–8.0), 9.2 (6.2–8.1), 3.9 (2.8–3.6)], II 21.3 (15.0–18.9) [5.9 (4.0–5.1), 2.0 (1.5–1.9), 5.7 (4.1–5.0), 5.2 (3.5–4.6), 2.5 (1.9–2.3)], III 13.1 (9.8–11.8) [3.9 (2.8–3.4), 1.6 (1.3–1.5), 3.1 (2.4–2.8), 2.9 (2.1–2.7), 1.6 (1.2–1.4)], IV 19.8 (14.5–17.9) [5.5 (4.0–4.9), 2.0 (1.5–1.7), 5.2 (4.1–4.8), 4.8 (3.3–4.5), 2.3 (1.6–2.0)]. Leg formula: 1243. Palpal claw with 9 (9–10) teeth. Spination (holotype from Madatory, India). Palp: 110, 000, 0000, 0000; legs: femur I 412, II 312, III 113, IV 011; patella I–IV 000; tibia I 2006, II 3004, III 0013, IV 0013; metatarsus I–II 2015, III 1015, IV 1014. Colouration: As described for the genus *Fecenia*. Sebastian and Peter (2009, plate 94) show a photo of female habitus. Copulatory organ: In epigyne AS clearly broader than PS (Figs 76, 111). AML strongly sclerotised. Epigynal muscle sigilla (EM) integrated in epigynal field. Female holotype with four slit sense organs (SO) on each side outside the epigynal field (EF) (Fig. 76), ♀ SB 982 from Sri Lanka with three SO on each side, all in EF and ♀ SB 118 from Thailand with one on each side outside EF (Fig. 71). In contrast to *Fecenia protensa*, folding of AS may be extending further anteriorly than AML (Figs 76, 111), but not always. Vulva with short (shorter than in all *Fecenia* species but *Fecenia macilenta*) and broad TSI (Fig. 77). SSI may be darker than in *Fecenia protensa* and with ca. 2 curves (Figs 78, 112). Primordial copulatory organ: Pre-epigyne: Very similar to *Fecenia protensa*, but lateral prongs of the “crown” narrower (Fig. 74, in Fig. 113 hard to recognise). Pre-vulva: Very similar to *Fecenia protensa* in having bulbous/spherical pre-receptacula (Figs 59, 67–68, 75), with centres of the latter being rather far away (more than three times the diameter of one pre-receptaculum). *Fecenia travancoria* is hard to distinguish from *Fecenia protensa* by the characters of the pre-vulva. In *Fecenia travancoria* the receptacula are rather oval in shape (Fig. 75), in *Fecenia protensa* round. Variation of copulatory organs: In ♀ SB 118 (Fig. 71) from Erawan, Thailand the distance between AS and AML is shorter than in holotype. In ♀ SB 118 (Fig. 71) and in ♀ SB 982 from Sri Lanka the folding of AS extending not as far anteriorly than in holotype (Fig. 76). The vulvae of the ♀♀ examined as well as the primordial copulatory organs of the s.a. ♀♀ showed no significant variation.

##### Remarks.

This species is very similar to *Fecenia protensa*. There are only fine differences in characters of the vulva (see diagnosis). Up to now, no intermediate forms concerning the shape of vulva have been found. Though it cannot be fully excluded, it seems rather unlikely that *Fecenia travancoria* is a junior synonym of *Fecenia protensa*. Generally, in *Fecenia* species the vulva shows less intraspecific variation than the epigyne. By now I consider *Fecenia travancoria* as valid species. But with more material from the southern Provinces of India, especially males, it may be possible to clarify this ‘difficult taxonomic case'.

##### Disribution.

India [Kerala Prov.], Sri Lanka, Thailand.

**Figures 79–82.**
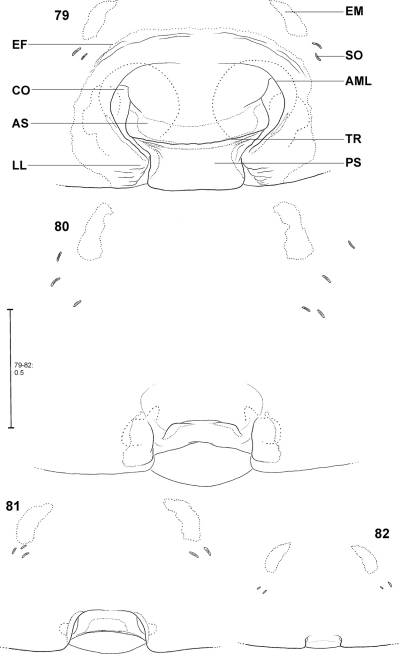
*Fecenia cylindrata*, ♀ epigyne/primordial epigyne, ventral view **79** ♀ SB 919 **81** s.a. ♀ SB 911, both from Bago Prov., Myanmar **80** s.a. ♀ SB535 from Champasak Prov., Laos **82** p.s.a. ♀ SB 937 from Luang Prabang Prov., Laos **79** Epigyne **80–81** Pre-epigyne **82** Pre-pre-epigyne. AML = Anterior margin of lateral lobe; AS = Anterior part of median septum; CO = Copulatory opening; EF = Epigynal field; EM = Epigynal muscle sigilla; LL = Lateral lobe; PS = Posterior part of median septum; SO = Slit sense organ; TR = Transversal edge/ridge of median septum.

**Figures 83–86. F17:**
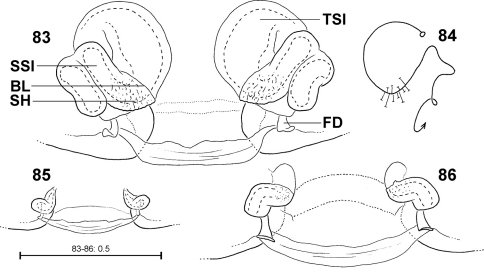
*Fecenia cylindrata*, ♀ vulva/pre-vulva, dorsal view **83–84** ♀ SB 919 **85** s.a. ♀ SB 911, both from Bago Prov., Myanmar **86** s.a. ♀ SB535 from Champasak Prov., Laos **83** Vulva **84** Course of internal duct system **85–86** Pre-vulva. BL = Borderline between SSI and TSI; FD = Fertilisation duct; SH = Spermathecal head; SSI = Strongly sclerotised section of internal duct system; TSI = Transparent section of internal duct system.

**Figures 87–90. F18:**
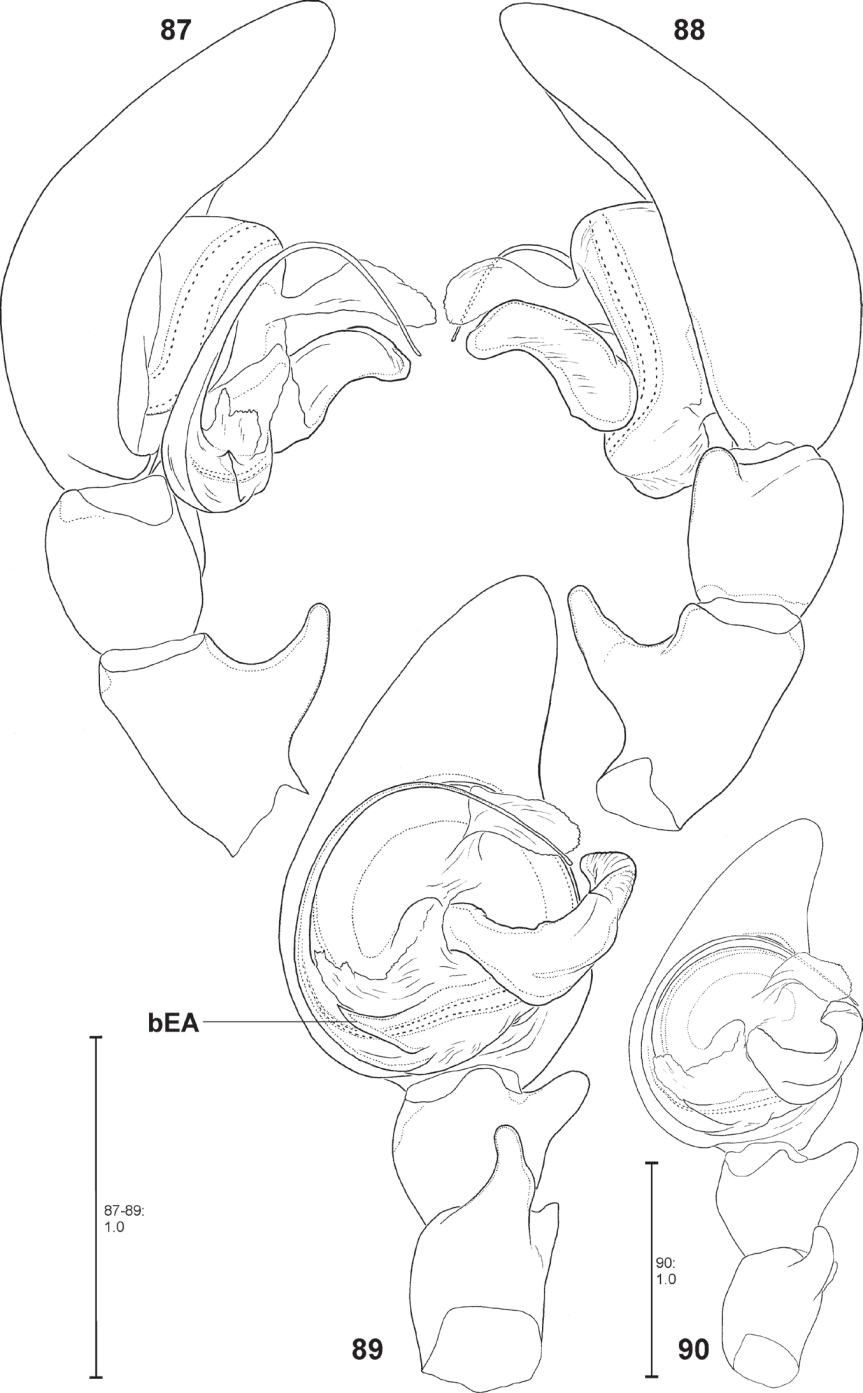
*Fecenia cylindrata*, ♂ palp **87–89** SB 928 from Bago Prov., Myanmar **90** SB 111 from Hainan, China **87** Prolateral view **88** Retrolateral view **89–90** Ventral view. Remark on Fig. 90: Details omitted, embolus slipped behind conductor. bEA = Basal embolus apophysis.

**Figures 91–94.**
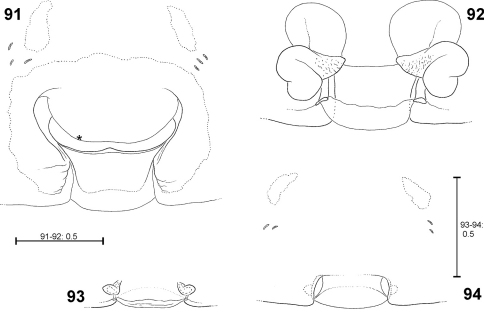
*Fecenia cylindrata*, ♀ copulatory organ/primordial copulatory organ **91–92** ♀SB 110 from Hainan, China **93–94** s.a. ♀SB 921 from Bago Prov., Myanmar **91** Epigyne, ventral view **92** Vulva, dorsal view **93** Pre-vulva, dorsal view. **94** Pre-epigyne, ventral view. Remark on Figs **91–92**: Details omitted. Asterisk indicates the folding, which divides the anterior from the posterior part of AS.

**Figures 95–101.**
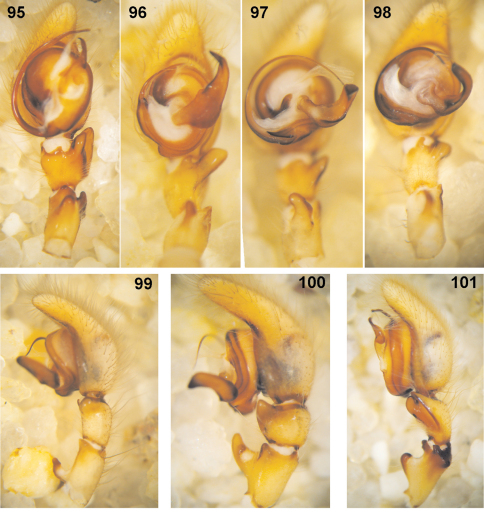
*Fecenia* spp., male palp **95, 101** *Fecenia macilenta* **96** *Fecenia ochracea* **97, 100** *Fecenia cylindrata* **98–99** *Fecenia protensa* **95, 101** SB 389 from Selangor Prov., Malaysia **96** SB 187 from Halmahera, Indonesia **97** SB 510 from Champasak Prov., Laos **100** SB 204 from Chaiyaphoom Prov., Thailand **98–99** SB 512 from Koh Chang, Thailand **95–98** ventral view **99–101** retrolateral view.

#### Fecenia cylindrata

Thorell, 1895

http://species-id.net/wiki/Fecenia_cylindrata

Figs 79 [Fig F17] [Fig F18] –94 97, 100 105–107 116–117 120–123

Fecenia cylindrata Thorell, 1895: 64 (Description of juveniles), [2 syntypes: juvenile syntype (SB 281, neither penultimate nor antepenultimate instar, thus sex unknown) from MYANMAR: Bago Prov.: Delta near Tharrawaddy (ca. 100 km NW of Yangon); 1884–1887, ded. E.W. Oates; NRS Thorell-Coll.-No. 70a., examined; other juvenile syntype from MYANMAR: Tanintharyi Prov.: Dawei, “on an island in Tavoy river”; 1884–1887, ded. E.W. Oates; type deposition unknown, maybe lost, thus not examined]; Thorell 1897: 263 (Description of ♂ and ♀); Pocock 1900: 212 (Description of ♀); Kulczyński 1908: 570; Reimoser 1936: 406; Lehtinen 1967: 234, figs 472–473 (Illustration of carapace, illustration of ♂); Levi 1982: 136, figs 80–82 (Illustration of ♂ and ♀); Murphy 1986: 65; Yang and Wang 1993: 29, figs 1–4 (Illustration of ♂ and ♀); Song et al. 1999: 397, figs 231O–Q (Illustration of ♂ and ♀); Wang and Yin 2001: 332, figs 1–4 (Illustration of ♂ and ♀).
Fecenia hainanensis Wang, 1990: 257, figs 1–3 (Description of ♀), [Holotype ♀ from CHINA: Hainan Province: Tongqian city, 18°30'N, 109°45'E; Liu leg. 01.VII.1984; HBI, not available on request, thus not examined, examined by Wang and Yin (2001)]; Wang and Yin 2001: 332 (Synonymy).

##### Additional material examined.

(10 ♂♂, 35 ♀♀, 5 s.a. ♂♂, 15 s.a. ♀♀, 1 p.s.a. ♀, 12 juvenile specimens). **CHINA:** **Hainan Prov.:** Mount Jainfeng; 20.IV.1990, ded. D.X. Song; 1 ♂ (SB 111), 1 ♀ (SB 110), Deeleman Coll. in RMNH. **MYANMAR:** **Sagaing Prov.:** Chattin Wildlife Sanctuary, Takontaing camp, 22°37'20"N, 95°31'52"E; J. Coddington & R.L.C. Baptista leg. 7–12.X.1998 by night; 2 ♀♀ (SB 182, 188), USNM. **Bago Prov.:** Palon; L. Fea leg. 1885–1889, “Viaggio in Birmania”; T. Thorell det. 20.X.1896; 2 ♂♂ (SB 289–290), 5 ♀♀ (SB 282–286), 1 s.a. ♀ (SB 287), 1 s.a. ♂ (SB 288), NRS Thorell-Coll.-No. 70b; 5 ♀♀ (SB 915–919), 3 s.a. ♀♀ (SB 910–912), 2 s.a. ♂♂ (SB 913–914), ZMH; 1 ♂ (SB 928), 4 ♀♀ (SB 929–932), 8 s.a. ♀♀ (SB 920–927), 2 s.a. ♂♂ (SB 933–934), 1 juv. (SB 935), ZMUC 5772. The following material has the same dates as above, but was checked via photos of copulatory organs kindly provided by P. Dankittipakul: 2 ♂♂ (SB 827–828), 5 ♀♀ (SB 822–826), MCSN. **THAILAND:** **Chiang Mai Prov.:** Doi Suthep N.P.; P. Dankittipakul leg.; 1 ♀ (SB 205), MHNG. Lamphun Prov.: Mae Tha Distr.: Doi Khuntan N.P., 800 m; P. Schwendinger leg. 22.IX.1994; 1 ♀ (SB 135), MHNG. **Loei Prov.:** Na-Haeo, field research station; J. Constant, K. Smets & P. Frootaart leg.15.–19.V.2003; 1 ♀ (SB 11), IRSN. **Chaiyaphoom Prov.:** Phu Kradung N.P., 1200-1300 m, flat plateau with mixed deciduous + pine + evergreen forest; P. Dankittipakul leg. 15.VIII.2006; 1 ♂ (SB 204), MHNG. **LAOS:** **Luang Prabang Prov.:** near Luang Prabang: Tham Sieng Mang, 19°54'09"N, 102°08'32"E, 270 m, sunny + dry area, low shrubs; P. Jäger & S. Bayer leg. 15.XI.2009; 1 ♀ (SB 485), SMF. Luang Prabang: Phou Si, 19°53'23"N, 102°08'04"E, 300 m, dry secondary forest in town, in shrubs; P. Jäger & V. Vedel leg. 12.XI.2004; 3 juvs (SB 938–940); P. Jäger leg. 25.III.2007; 1 s.a. ♀ (SB 62), 1 p.s.a. ♀ (SB 937); P. Jäger & S. Bayer leg. 14.XI.2009; 1 ♂ (SB 488, deformed, died during adult moult), all SMF. SE of Luang Prabang: Nam Khan, Xieng Ngeun Distr., Ban Keng Koung, 19°40'963"N, 102°18'442"E, ca. 370 m, along river bank; P. Jäger leg. 24.II.2008; 1 juv. (SB 936), SMF. **Champasak Prov.:** Muang Bachieng: That Paxuam, 15°10'35"N, 105°55'21"E, 200 m, secondary forest; P. Jäger & S. Bayer leg. 25.XI.2009; 1 ♀ (SB 318), 2 juvs (SB 40–401), SMF. Ban Lak 38, That Fane, 15°11'03"N, 106°07'37"E, 950 m, coffee plantation; P. Jäger leg. 11.–16.III.2010; 1 ♀ (SB 528), 1 s.a. ♀ (SB 535), 3 juvs (SB 527, 532–533), SMF. Near Pakse: Ban Ke, 15°07'57"N, 105°48'54"E, 100 m, dry shrubs; P. Jäger & S. Bayer leg. 27.XI.2009, by night; 1 juv. (SB 351), SMF. Muang Pathoumphone: Vat Phou Salao, 15°05'39"N, 105°48'35"E, 150 m, dry bed of stream, dry shrubs; P. Jäger & S. Bayer leg. 24.XI.2009, by night; 3 ♀♀ (SB 48–487, 514), 3 juvs (SB 349, 398, 526), SMF. Ban Nog Hoy, N slope of Phou Malong, 15°03'14"N, 105°49'07"E, 115 m, dry bed of stream, dry shrubs; P. Jäger leg. 23.XI.2009; 2 ♂♂ (SB 50–510), 1 ♀ (SB 511), 1 s.a. ♀ (SB 420), 2 juvs (SB 39–397), SMF. Ban Tha Hou, 14°46'10"N, 105°59'35"E, 130 m, dry forest, near summit of a prominent hill; P. Jäger & S. Bayer leg. 22.XI.2009; 2 ♀♀ (SB 513, 525), SMF.

##### Diagnosis.

Females distinguished from other *Fecenia* species except *Fecenia protensa* and *Fecenia travancoria* by having anteriorly converging anterior margins of lateral lobes (AML) partly surrounding epigynal pit; distinguished from *Fecenia protensa* and *Fecenia travancoria* by the even and unfolded anterior part of median septum (AS) and by transverse edge of median septum (TR) lacking distinct notch (Figs 79, 105). In vulva transparent section of internal duct system (TSI) larger than strongly sclerotised section (SSI) (Figs 83, 92, 106). Males distinguished from other *Fecenia* species except *Fecenia protensa* by having short (at most half as long as width of palpal tibia) RTA; distinguished from *Fecenia protensa* in having RTA, which is longer than broad, and ventral patellar apophysis (VPA) arising centrally on patella (Fig. 88). Median apophysis (MA) running almost in transversal plane (Figs 87–88, 100). Embolus (E) with pointed basal apophysis (bEA) (Fig. 89).

**D**

##### escription.

MALE:Body and eye measurements. Carapace length 3.6–4.4, carapace width 2.2–2.8, anterior width of carapace 1.6–1.9, opisthosoma length 4.8–6.5, opisthosoma width 1.9–2.5. Eyes: AME 0.23–0.29, ALE 0.17–0.22, PME 0.17–0.22, PLE 0.18–0.21, AME–AME 0.16–0.23, AME–ALE 0.09–0.14, PME–PME 0.17–0.25, PME–PLE 0.28–0.35, AME–PME 0.12–0.16, ALE–PLE 0.10–0.15, clypeus height at AME 0.30–0.41, at ALE 0.24–0.35. Measurements of palp and legs. Palp 4.0–5.0 [1.6–2.0, 0.6–0.7, 0.4–0.5, 1.4–1.8], I 35.1–48.3 [9.5–12.9, 1.5–2.1, 9.3–13.6, 10.5–14.9, 4.3–4.8], II 16.6–22.0 [4.3–5.9, 1.3–1.6, 4.6–6.4, 4.3–5.6, 2.1–2.5], III 8.7–11.8 [2.5–3.4, 1.0–1.2, 2.0–2.9, 2.1–2.8, 1.1–1.5], IV 15.0–20.2 [4.2–5.4, 1.2–1.6, 3.8–5.6, 4.0–5.4, 1.8–2.2]. Leg formula: 1243. Copulatory organ: Retrolateral patellar apophysis (RPA) mostly more clearly visible (Fig. 89) than in *Fecenia protensa* and *Fecenia ochracea*. Tip of MA in ca. 2:30–3:00-o'clock-position, MA shorter than width of tegulum (T) (Figs 89–90, 97) and in pro- and retrolateral view curved distally (Figs 87–88, 100). E very slim, especially distally, arising in ca. 7:30-o'clock-position on T and clearly longer than width of T (Figs 89–90, 97). The latter almost round. Membranous process of tegulum (MP) reaches at most to 8:30-o'clock-position (Figs 89–90, 97). Conductor (C) longer than in *Fecenia ochracea* and *Fecenia protensa*, shorter than in *Fecenia macilenta*, arises medially (or slightly shifted retrolaterally) in upper third of T (Figs 89–90). Cymbium in relation a bit longer than in all other *Fecenia* species (Figs 87–88, 97, 100). Scopula dorsally on cymbium slightly less developed than in other *Fecenia* species.

FEMALE: Body and eye measurements. Carapace length 3.7–7.2, carapace width 2.2–4.2, anterior width of carapace 1.7–3.2, opisthosoma length 7.3–13.0, opisthosoma width 3.5–6.0. Eyes: AME 0.20–0.27, ALE 0.15–0.24, PME 0.16–0.22, PLE 0.16–0.22, AME–AME 0.18–0.34, AME–ALE 0.07–0.18, PME–PME 0.20–0.29, PME–PLE 0.33–0.54, AME–PME 0.14–0.27, ALE–PLE 0.12–0.27, clypeus height at AME 0.27–0.54, at ALE 0.25–0.48. Measurements of palp and legs. Palp 3.8–6.5 [1.3–2.3, 0.6–1.1, 0.7–1.1, 1.2–2.0], I 22.7–39.3 [6.0–10.8, 1.6–2.9, 6.3–10.8, 6.1–10.7, 2.7–4.1], II 13.1–23.7 [3.3–6.4, 0.9–2.3, 3.7–6.8, 3.1–5.7, 1.6–2.5], III 7.5–14.1 [2.2–4.1, 0.9–1.9, 1.8–3.4, 1.6–3.1, 1.0–1.6], IV 12.3–21.4 [3.3–5.9, 1.2–2.3, 3.5–6.2, 2.9–4.9, 1.4–2.1]. Leg formula: 1243. Palpal claw with 8–11 teeth. Spination (remaining immature syntype from Tharrawaddy in poor condition! Spination of female SB 285 from Palon, Birma (Myanmar) listed instead). Palp: 110, 000, 0100, 2004 (spines on tibia and tarsus with only half the size as those of femur!); legs: femur I 300(200), II 210, III 221(111), IV 010; patella I–IV 000; tibia I 0006(1005), II 2004(3005), III–IV 0024; metatarsus I 2015(2016), II–III 2015, IV 1018. Copulatory organ: Anterior part of AS divided from posterior part of AS by a differently developed folding (asterisk in Fig. 91). AS broader than PS (Fig. 79). Epigynal muscle sigilla (EM) clearly outside epigynal field (Figs 79, 91). Slit sense organs (SO) outside epigynal field. Vulva with large and broad TSI (Fig. 83), mostly larger than SSI. The latter with longer duct than in *Fecenia protensa*, *Fecenia travancoria* and *Fecenia ochracea*, with 3–4 curves (Fig. 84). Border line (BL) between TSI and SSI of vulva in ca. 7:00-8:00 o' clock position (Figs 83, 92, 106). Primordial copulatory organs: Pre-epigyne: TR continuous (Fig. 81, 107), slightly recurved. AML anteriorly bent sharply, running medially and (almost) meeting each other (Figs 81, 94, 107). Epigynal field not or only poorly developed, EM far outside epigynal field. Pre-pre-epigyne: AML similar to pre-epigyne, TR hardly recognisable (Fig. 82). Pre-vulva: Pre-receptacula with lateral extension (Figs 85, 93). Distance between centres of pre-receptacula more than three times the diameter of one pre-receptaculum (Figs 85, 93). Variation of copulatory organs: In males position of VPA may shift retrolaterally (Fig. 90). Direction of MA (Figs 89, 90, 97) may vary. Anterio-medial section of C differs among specimens examined (Figs 89, 90, 97). RTA in some specimens basally broader (Fig. 90). In females the folding which divides (or partly divides) the anterior from the posterior part of AS differently developed (Figs 79, 91). TR rarely with a very small, flat and indistinct notch (Fig. 91). AS and PS in some specimens less broad than in others. Number of SO among specimens varying without geographical dependence. Anterio-lateral section of SSI may differ in shape (Figs 83, 92). Pre-epigynes differing in length and direction of AML (Figs 81, 94), further in shape of TR (Figs 81, 94). The most frequent pre-epigyne type is the one of SB 911 (Fig. 81). Number of SO varying strongly. SB 535 from Champasak Province, Laos (Fig. 80) is an exception, which is discussed explicitly (see discussion below).

The pre-vulvae differ only slightly (Figs 85, 93). SB 535 (Fig. 86) is an exception, which is discussed explicitly (see discussion below).

##### Remarks.

Thorell (1895) described this species based on juvenile types. Two years later Thorell himself redescribed this species based on ♂♂ and ♀♀ recorded just ca. 70 km away from type locality Tharrawaddy (Thorell 1897). This material is deposited in NRS, ZMH and MCSN and was examined (see material list above). Moreover, to date no other *Fecenia* species than the one described above had been found in Myanmar. For that reason there are no doubts about the identiy of *Fecenia cylindrata*.

*Fecenia hainanensis* Wang, 1990 was synonymised with *Fecenia cylindrata* by Wang and Yin (2001). The female holotype from Tonqian, Hainan Province, China was not available on request. According to the illustrations in Wang (1990), which are not very detailed, it is more likely that his *Fecenia hainanensis* was in fact conspecific with *Fecenia cylindrata*. The specimens from Hainan checked in the present study are considered belonging to *Fecenia cylindrata*, though there are slight differences (see variation of copulatory organs in the description of *Fecenia cylindrata*). More material from Hainan and also from regions of South East China and Northern Vietnam is necessary to assess the consistency of those slight differences among the different specimens. At the moment *Fecenia hainanensis* is regarded as junior synonym of *Fecenia cylindrata*.

##### Distribution.

China, Myanmar, Laos, Thailand.

**Figures 102–115.**
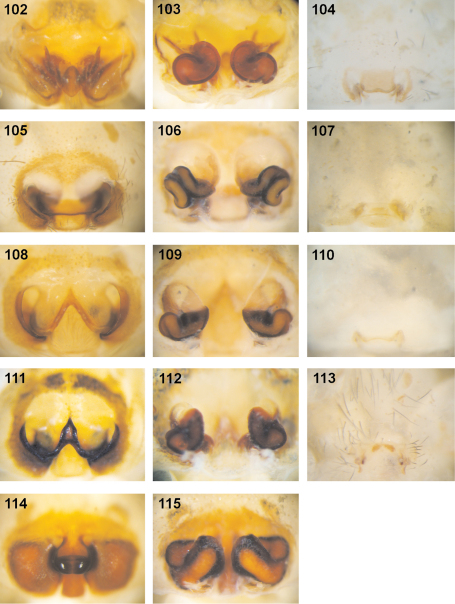
*Fecenia* spp., female copulatory organs/primordial copulatory organs **102–104** *Fecenia ochracea* **105–107** *Fecenia cylindrata* **108–110** *Fecenia protensa* **111–113** *Fecenia travancoria* **114–115** *Fecenia macilenta* **102–103** ♀ SB 668 from New Guinea **104** s.a. ♀ SB 540 from New Britain, Papua New Guinea **105–106** ♀ from Loei Prov., Thailand **107** s.a. ♀ from Palon, Bago Prov., Myanmar **108–109** ♀ SB 215 from Songkhla Prov., Thailand **110** s.a. ♀ holotype of *Fecenia protensa* (SB 620) from Nicobar Islands. **111** ♀ holotype of *Fecenia travancoria* (SB 403) from Kerala Prov., India **112–113** ♀ SB 982 from Sri Lanka (remark on 113: photo of exuviae of subadult instar of same specimen as in 112) **114–115** ♀ SB 124 from Sumatera Barat Prov., Indonesia **102, 105, 108, 111, 114** ♀ epigyne, ventral view **103, 106, 109, 112, 115** ♀ vulva, dorsal view **104, 107, 110, 113** pre-epigyne of s.a. ♀.

**Figures 116–120.**
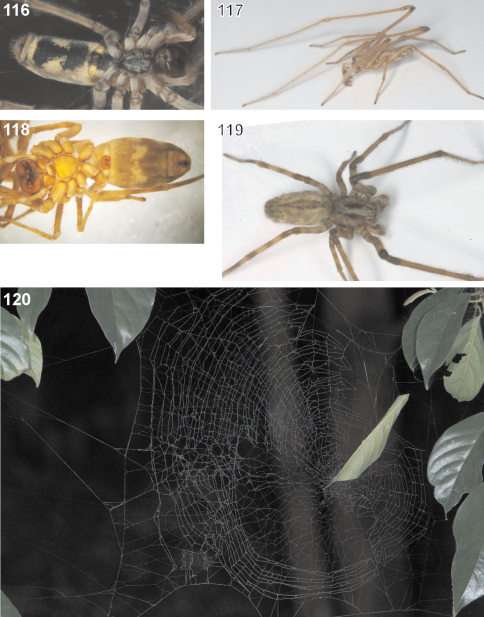
*Fecenia* spp., habitus, web **116–117, 120** *Fecenia cylindrata*, ♀ SB 486 (**116**), ♂ SB 509 (**117**) from Champasak Prov., Laos, web (**120**) from Xishuangbanna, China **118** *Fecenia ochracea*, ♀ SB 161 from Auki, Solomon Islands **119** *Fecenia protensa*, ♀ SB 256 from Bali, Indonesia **116, 118** Habitus, ventral view **117** Habitus, dorso-lateral view **119** Habitus, dorsal view **116–117, 119** Photos by Peter Jäger **120** Photo by Jeremy Miller.

**Figures 121–123.**
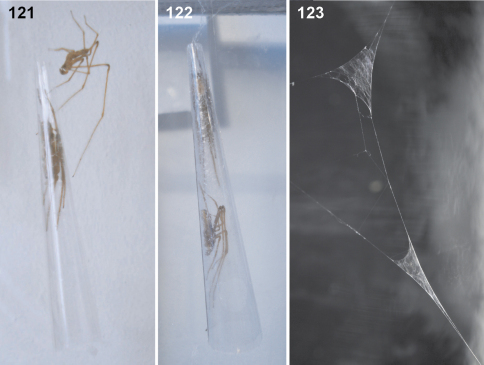
*Fecenia cylindrata*, ♂ (SB 510) and ♀ (SB 487) from Champasak Prov., Laos, mating behaviour, sperm web **121** ♂ stroking behaviour upon the retreat of the female **122** ♂ and ♀ together in the retreat **123** ♂ sperm webs (SB 510) **123** Photo by Peter Jäger.

## Discussion

### Characteristics of the pre-epigyne

The pre-pre-epigyne (antepenultimate instar), although hardly useful for species determination, may bear important information. In some *Fecenia* species both pre-subadult and subadult females were available for examination. A continuous developmental trend from pre-pre-epigyne (p.s.a. ♀♀) to the epigyne of adults can be traced (e.g. Figs 19–22 for *Fecenia ochracea*, Figs 79–82 for *Fecenia cylindrata*). Sierwald (1989) showed that in most of the American Pisauridae even more primordial epigyne stages exist. In *Pisaurina mira* (Walckenaer, 1837) up to five stages with differently developed primordial copulatory organs (which Sierwald denominated as “anlagen”) occur. Gradually from earlier to later stages the anlagen resemble more and more the adult. The changes from penultimate instar to adult constitute the largest developmental step as the shapes of pre-epigynes and adult epigynes differ the most. The number of primordial stages in *Pisaurina mira* varies between three and five (Sierwald 1989). Interestingly, in specimens with only three primordial stages, the anlagen of the antepenultimate and penultimate instars were less developed and differentiated. Anyway, these specimens moult following their third anlage to “normal” mature females (Sierwald 1989). The total number of juvenile stages varies in Pisauridae. For example in *Dolomedes triton* (Walckenaer, 1837) the number ranges from 10 to 15 in males and 9 to 15 in females (Zimmermann and Spence 1998).

The present study reveals the occurrence of a different developmental stage of the pre-epigyne (penultimate instar) in the pseudo-orbweaver *Fecenia cylindrata* (Fig. 80). The following preliminary considerations may explain this phenomenon:

In insects a juvenile hormone (JH) regulates the development of the larva throughout the several moults up to the imago. Following Wigglesworth (1952), a controlled hormone balance between JH and prothoracotrope hormone is essential for regular development of the bug *Rhodnius prolixus* Stål, 1859. From 1^st^ to 4^th^ stage larva the concentration of JH decreases more or less continuously, but from the 4^th^ to 5^th^ stage the decrease is much stronger and from 5^th^ stage to imago JH is completely absent (Wigglesworth 1952). It is likely that JH exists in spiders, too (Webber 2005). Prothoracotrope hormone does not exist in spiders, but instead of this it is possible, that another, equivalent hormone exists.

On the other hand it is known from spiders that the number of moults, and thus the number of instars, to reach maturity may differ, for example in Pisauridae (see above). In *Latrodectus mactans* (Fabricius, 1775) the number of instars varies from 7 to 9 depending on food supply (Deevey 1949). From particular species of *Stegodyphus* Simon, 1873 it is also known that maturity is reached after different numbers of moults in different specimens examined, irrespective of their sex (Kullmann et al. 1972, Kraus and Kraus 1988). Furthermore, Kraus and Kraus (1988) state that the enormous size variation in species of *Stegodyphus* seems to be caused mainly by this flexibility. At least in the species *Fecenia ochracea* and *Fecenia cylindrata*,the size variation is high. This becomes obvious by their carapace-length size ranges (see respective descriptions). It is possible that in *Fecenia* the number of moults required to reach maturity differs intraspecifically, too. Considering that the number of stages of immature females with differently developed primordial copulatory organs varies in Pisauridae (see above), a family also belonging to the Lycosoidea (Griswold et al. 2005), it is not unlikely that this applies to the pseudo-orbweavers too. A pre-epigyne of a s.a. ♀ of the 6^th^ instar would then most likely differ from the one of an 8^th ^instar.

In *Fecenia* it seems to be rare, that the pre-epigyne of a particular subadult female differs from the ones of the others belonging to the same species. But, anyway, as the example of the subadult female of *Fecenia cylindrata* (Fig. 80) shows, this phenomenon may appear. In such a case additional consideration concerning the identification of subadults is necessary. Does the respective subadult female fit into a conceivable developmental continuum for the species in question? This is, of course, much easier if several “regularly” developed s.a. ♀♀ and/or p.s.a. ♀♀ are available. As the pre-epigyne of a “further developed” s.a. ♀ most likely resembles more an adult epigyne than a “regularly developed” one does, it should not be too difficult to identify it. Thus, in *Fecenia* the pre-epigynal characters apparently are species-specific (pre-epigynes, take notice; this must not inevitably mean that this applies also to the pre-pre-epigynes or other primordial epigynes of instars below subadult females!). Following the studies of (Sierwald (1987, 1989) the pre-epigynes of the Pisauridae species examined seem to be specific, too. Hence, it is justified to use the pre-epigyne as a tool for identification.

### Validity of characters in *Fecenia*.

Somatic characters are not useful for species determination in *Fecenia*. Colouration and spination, for example, are highly variable intraspecifically. Figures 69 and 82 in Levi (1982) suggest that species discrimination between *Fecenia ochracea* and *Fecenia cylindrata* via colouration of the ventral surface of the opisthosoma is possible. According to the present study, this cannot be confirmed. Species identification is only possible by checking the copulatory organs.

### Remarks on spination

In the description of the genus *Fecenia* above a characteristic aspect of the spination pattern on the tibiae is mentioned. This may be explained by the life style of the pseudo-orbweavers. *Fecenia* is the only spider genus in which all representatives spend at least 95% of their lifetime in a very narrow enrolled-leaf retreat or cone retreat in early juveniles. In Araneidae there are several genera including species, that have similar lifestyles, e.g. *Acusilas*, *Cyclosa*, *Neoscona*, *Araneus*, *Cyrtophora*, also in Theridiidae, e.g. *Parasteatoda simulans* (Thorell, 1875). In any case, there is no genus in which all representatives use enrolled leaves as a retreat. Furthermore, in representatives of the families mentioned above the leaf-retreat is never as narrow (in relation to body size) as in *Fecenia*. A pseudo-orbweaver enters its retreat always with its opisthosoma first. The patellae and tibiae have the most intensive contact with the inner walls of the leaf retreat. As the legs are prograde with leg pairs I–II held anteriorly and III–IV posteriorly it becomes obvious that in the first two leg pairs the retrolateral and in the last two leg pairs the prolateral spines on the tibiae would be an impediment while moving inside the retreat. Perhaps in the course of the evolution of this genus, specimens with shorter spines or even no more spines at these respective positions were preferred? Like in *Psechrus* the patellae completely lack spines (Lehtinen 1967). This characteristic aspect of the tibial spination pattern in *Fecenia* may be an adaptation to this special life style. It would be interesting to check if the tibial spination pattern of species from the Araneidae and Theridiidae genera listed above using enrolled leaves, differ from the ones with different lifestyles. But in contrast to Psechridae in Araneidae and Theridiidae the spines are in any case not so prominent in comparison to leg diameter.

## Supplementary Material

XML Treatment for Fecenia

XML Treatment for Fecenia ochracea

XML Treatment for Fecenia macilenta

XML Treatment for Fecenia protensa

XML Treatment for Fecenia travancoria

XML Treatment for Fecenia cylindrata
